# Extracellular vesicles as emerging platforms for modulating innate immune responses in sepsis-associated acute lung injury

**DOI:** 10.3389/fimmu.2026.1793559

**Published:** 2026-04-17

**Authors:** He Yan, Lin Zhang, Xuejiao Lv, Tingting Guo, Qi Wang, Jie Zhang

**Affiliations:** Department of Respiratory and Critical Care Medicine, the Second Hospital of Jilin University, Changchun, China

**Keywords:** acute lung injury, drug delivery systems, extracellular vesicles, immunity, innate, nanoparticles, sepsis

## Abstract

Sepsis-associated acute lung injury (ALI) remains a major challenge in intensive care units, characterized by dysregulated innate immune responses that drive both excessive inflammation and subsequent immunosuppression. In recent years, extracellular vesicles (EVs) and EV-inspired biomimetic nanosystems have attracted increasing attention as candidate platforms for modulating immune imbalance in ALI. This review summarizes recent advances in understanding the immunopathological mechanisms underlying sepsis-associated ALI, including macrophage polarization imbalance, excessive neutrophil extracellular trap (NET) formation, dendritic cell functional exhaustion, and dysregulation of key signaling pathways such as TLR4, NLRP3 inflammasome, and cGAS–STING. We further discuss how naturally derived EVs and engineered EV-mimetic carriers may influence these pathogenic processes through the delivery of bioactive cargoes, drawing primarily from preclinical observations. In addition, current strategies for pulmonary-targeted delivery, EV engineering approaches, and major translational considerations, including biosafety, manufacturing standardization, and quality control, are critically evaluated. Although most available evidence derives from preclinical studies, EV-based biomimetic nanosystems represent a promising research direction that may complement existing anti-inflammatory strategies by integrating immune modulation, inflammation control, and tissue repair. Continued mechanistic investigation and clinically relevant validation will be essential for determining their therapeutic feasibility in sepsis-associated ALI.

## 1 Introduction

Sepsis is a life-threatening syndrome characterized by dysregulated host responses to infection. The clinical course of sepsis is highly heterogeneous and frequently involves a transition from an early hyperinflammatory phase to a later stage of immune suppression, commonly referred to as immunoparalysis. These dynamic immune alterations are particularly relevant in the lung, one of the earliest and most frequently affected organs during sepsis (1, 2). Pulmonary involvement can rapidly progress to acute lung injury (ALI) or its severe manifestation, acute respiratory distress syndrome (ARDS), which remains a leading cause of sepsis-related mortality (3, 4). Epidemiological data indicate that more than 30 million individuals develop sepsis each year worldwide. Among critically ill patients, approximately 30–50% eventually develop ALI or ARDS, with even higher incidence observed in elderly individuals and patients with underlying comorbidities (1, 3, 5). Large-scale clinical studies, including REMAP-CAP and MARS, have reported a 28-day mortality rate of 30–45% in patients with sepsis-associated ALI, whereas in-hospital mortality in intensive care units (ICUs) can exceed 50% (6).

Beyond its high mortality, sepsis-associated ALI is closely associated with substantial healthcare resource utilization and long-term health burdens (7–9). Clinical evidence indicates that ALI markedly prolongs ICU and hospital length of stay and increases the risk of ventilator-associated pneumonia, ICU-acquired weakness, and cognitive impairment (10). Importantly, even among survivors discharged from hospital, persistent pulmonary dysfunction and reduced quality of life remain common, reflecting a considerable long-term “survivor burden” (7).

Despite continuous advances in modern intensive care, current treatment of sepsis-associated ALI remains largely supportive and lacks specific targeted interventions. Widely adopted approaches, including mechanical ventilation, restrictive fluid management, and infection control, can stabilize patients but do not directly address the underlying immune-mediated inflammatory injury (11, 12).

Several immunomodulatory strategies have been explored to mitigate excessive inflammatory responses in sepsis. These approaches include glucocorticoids (11), interleukin-1 (IL-1) blockade using agents such as anakinra (13), and targeted cytokine modulation strategies directed at pathways such as interleukin-6 (IL-6) signaling (12). However, clinical trials evaluating these therapies have yielded heterogeneous and often conflicting outcomes, partly due to disease heterogeneity, variability in immune status, and differences in treatment timing. In addition, excessive immune suppression may increase susceptibility to secondary infections and contribute to sepsis-associated immunoparalysis (14). These limitations highlight the urgent need to develop novel therapeutic strategies capable of restoring immune balance while preserving host defense mechanisms.

The initiation and progression of sepsis-associated ALI are closely linked to dynamic dysregulation of innate immune responses. Compared with other inflammatory lung injury models, sepsis displays several distinctive immunopathological features, including a transition from early hyperinflammation to late immunosuppression, marked temporal heterogeneity in immune activation, and substantial inter-individual variability influenced by age, comorbidities, and pathogen characteristics. These features complicate therapeutic intervention and highlight the need for approaches capable of modulating immune responses across different disease stages. During the early phase of disease, neutrophils, macrophages, and dendritic cells (DCs) become excessively activated and amplify inflammatory cascades that damage alveolar structures. As the disease progresses, these immune cells may undergo functional exhaustion, leading to impaired antimicrobial defense and immunoparalysis (14–16). For instance, pulmonary neutrophils exhibit abnormal accumulation accompanied by enhanced release of neutrophil extracellular traps (NETs), while macrophage polarization may shift toward an M2-dominant phenotype characterized by elevated expression of immunosuppressive mediators such as interleukin-10 (IL-10) and programmed death-ligand 1 (PD-L1) (16) Both the number and functional capacity of DCs decline, resulting in impaired antigen presentation (17). These findings suggest that balanced regulation of innate immune responses represents an important therapeutic direction in ALI.

Extracellular vesicles (EVs) are nanoscale vesicles derived from immune cells or stem cells that possess intrinsic signaling and delivery capabilities. In recent years, they have attracted increasing attention as potential therapeutic candidates for immune imbalance–associated diseases such as ALI (15, 18, 19). Emerging evidence suggests that EVs derived from mesenchymal stem cells (MSCs) or other immunoregulatory cells may influence innate immune cell function through the transfer of bioactive molecules, including miRNAs) and proteins (12, 20).

In addition, biomimetic engineering strategies, including membrane fusion and membrane coating, have been explored to improve the pulmonary accumulation and structural stability of EVs, thereby facilitating the delivery of immunomodulatory factors (21, 22). Building on these advances, EV-based systems have been proposed as potential platforms for immune modulation in sepsis-associated ALI.

Based on this background, this review summarizes current insights into immune dysregulation in sepsis-associated ALI and discusses emerging strategies involving EVs and biomimetic EV-based systems. Particular emphasis is placed on innate immune regulation, biomimetic engineering approaches, delivery strategies, and the key challenges for clinical translation, with the aim of providing an integrated framework linking mechanistic understanding with therapeutic development.

## 2 Pathophysiological mechanisms of sepsis-induced ALI

### 2.1 Inflammatory cascade and pulmonary endothelial injury

**Figure 1:**
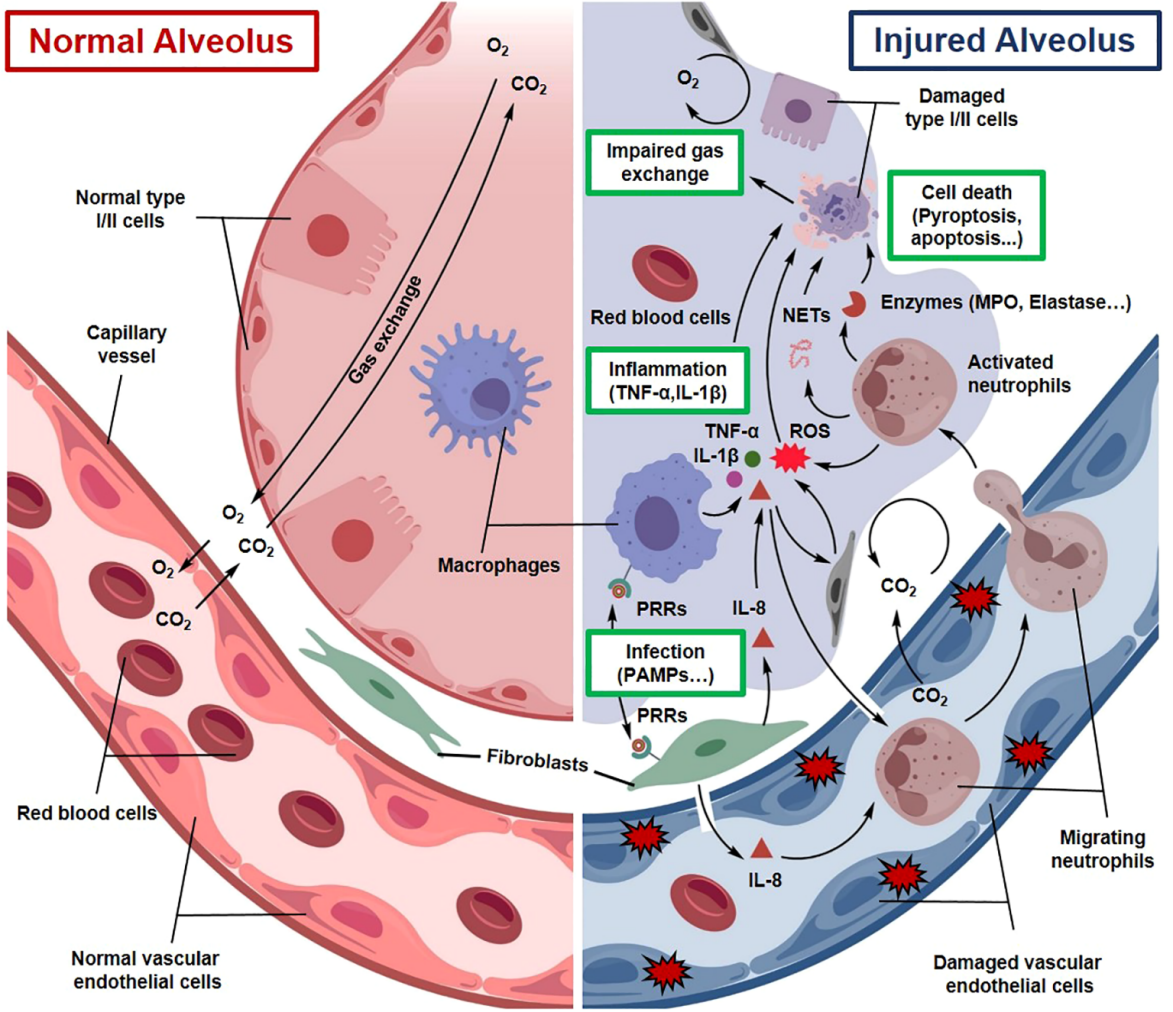
Schematic illustration of the pathogenesis of sepsis-associated ALI. Sepsis triggers a SIRS, leading to a “cytokine storm” characterized by the release of TNF-α, IL-1β, and IL-6. This inflammatory cascade causes pulmonary endothelial injury, increased vascular permeability, and alveolar flooding. Concurrently, immune cell infiltration and pyroptosis of alveolar epithelial cells further exacerbate tissue damage and respiratory dysfunction.

Sepsis-induced ALI is initiated by systemic inflammatory response syndrome (SIRS), which results from an excessive immune reaction to infection and is characterized by the large-scale release of proinflammatory mediators, commonly referred to as a “cytokine storm” (**Figure 1**) (23). During the early stage of sepsis, pathogen-associated molecular patterns (PAMPs) are recognized by host pattern recognition receptors (PRRs), leading to activation of downstream signaling pathways and rapid production of proinflammatory cytokines, including tumor necrosis factor-α (TNF-α), interleukin-1β (IL-1β), and IL-6 (24). Concurrently, activation of the NLRP3 inflammasome downstream of Toll-like receptor 4 (TLR4) signaling further amplifies inflammatory cascades and induces pyroptotic cell death (25).

Elevated concentrations of these cytokines directly impair pulmonary endothelial cells, disrupting intercellular junctions and increasing capillary permeability (26). Loss of endothelial barrier integrity allows plasma proteins and inflammatory cells to infiltrate the alveolar space, leading to pulmonary edema and worsening respiratory dysfunction (26). In addition, endothelial cell activation promotes leukocyte adhesion and transmigration, thereby intensifying inflammatory responses within the lung microenvironment (27).

Neutrophils are extensively recruited to the lung during sepsis-associated ALI and contribute substantially to alveolar epithelial and endothelial injury. Through degranulation, neutrophils release cytotoxic enzymes such as myeloperoxidase (MPO) and elastase and generate large amounts of reactive oxygen species (ROS), which directly damage the alveolar–capillary barrier and exacerbate pulmonary edema and hypoxemia (23). NETs further contribute to tissue injury. Recent studies have shown that NETs can activate the stimulator of interferon genes (STING) signaling pathway, promoting immunothrombosis and aggravating pulmonary endothelial damage (28).

Inflammatory stress also induces mitochondrial dysfunction, resulting in excessive ROS generation and subsequent activation of the NLRP3 inflammasome, which ultimately triggers pyroptosis (29). Pyroptosis represents an inflammatory form of programmed cell death that plays an important role in endothelial barrier disruption and ARDS progression (25). Mechanistically, inflammasome activation, particularly NLRP3, leads to caspase-1 activation and cleavage of gasdermin D (GSDMD), forming membrane pores that facilitate the release of proinflammatory cytokines such as IL-1β and IL-18. These processes amplify inflammatory signaling, promote immune cell recruitment, and further damage the pulmonary vascular barrier. In sepsis-associated ALI, pyroptosis of alveolar epithelial and endothelial cells therefore contributes to increased vascular permeability, alveolar flooding, and progressive impairment of gas exchange, ultimately accelerating disease progression (30).

### 2.2 Role of innate immune dysregulation in ALI

#### 2.2.1 Macrophage plasticity and polarization (M1/M2 polarization)

Macrophages exhibit marked plasticity in sepsis-associated ALI and can transition between the proinflammatory M1 and anti-inflammatory M2 phenotypes in response to microenvironmental signals (31). M1 polarization is primarily regulated by signaling pathways involving TLR4/NF-κB, hypoxia-inducible factor-1α (HIF-1α), and the glycolytic enzyme pyruvate kinase M2 (PKM2), whereas M2 polarization is associated with regulatory pathways such as SIRT1 and STAT6 that support anti-inflammatory and tissue-reparative function (32, 33). During the early stage of sepsis, M1 macrophages predominate and release large amounts of proinflammatory cytokines, including IL-1β and TNF-α, thereby amplifying inflammatory responses and promoting tissue injury (34–36). In contrast, M2 macrophages facilitate the resolution of inflammation and tissue reconstruction by secreting anti-inflammatory mediators and molecules that promote tissue repair (37). Recent studies have suggested that certain natural compounds, such as acacetin, can promote M2 polarization and thereby alleviate pulmonary inflammation and tissue injury, highlighting the potential of plant-derived interventions in sepsis-associated ALI (38). In addition, modulation of mitochondrial homeostasis and oxidative stress, such as SIRT3-mediated deacetylation of Opa1, has been shown to suppress M1 skewing, enhance M2 functionality, and improve the pulmonary immune microenvironment (39).

**Figure 2:**
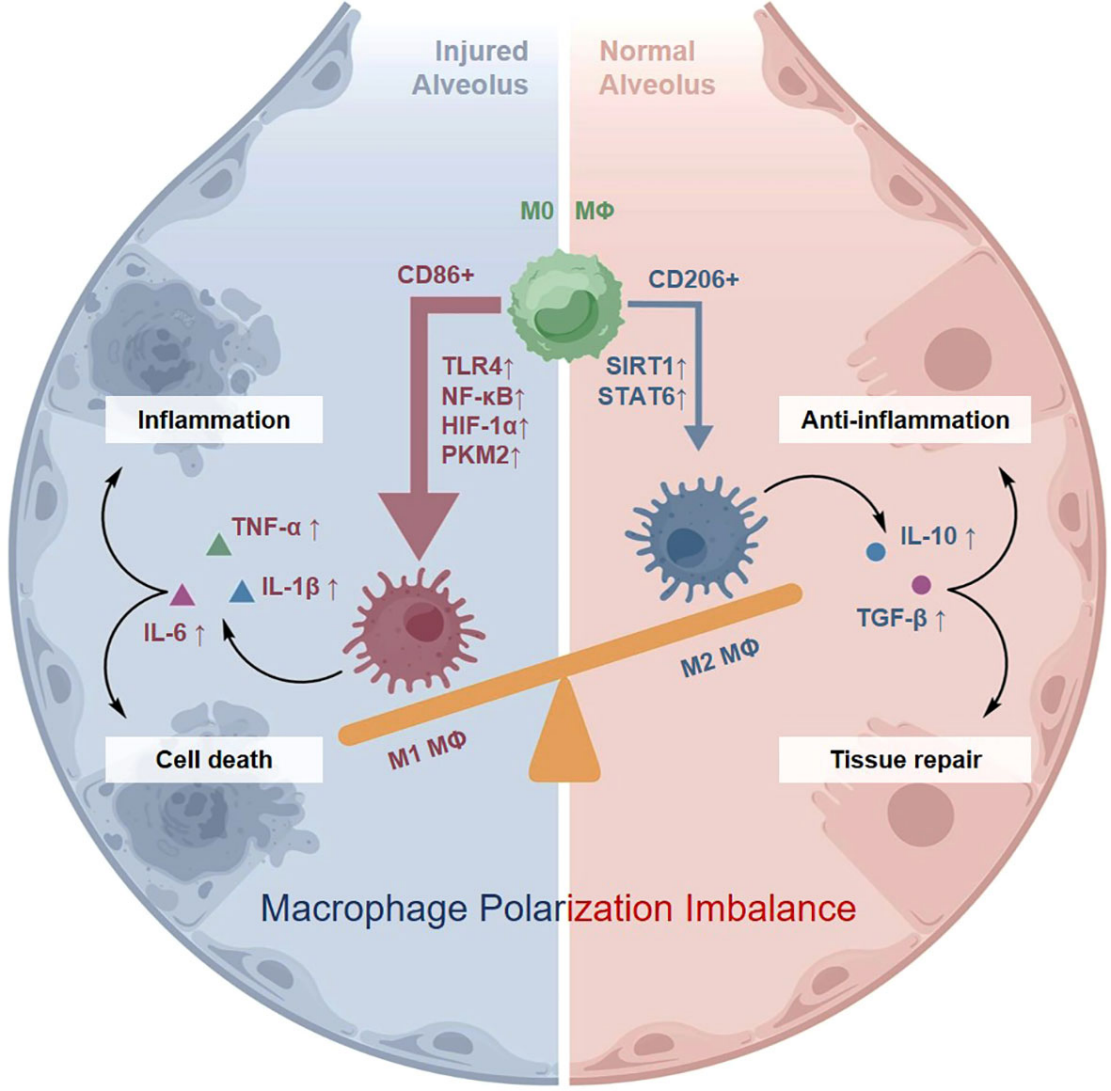
Bidirectional roles of macrophage polarization imbalance in ALI. Macrophages exhibit plasticity, transitioning between the pro-inflammatory M1 phenotype (induced by TLR4/NF-κB signaling) and the anti-inflammatory M2 phenotype (regulated by STAT6/SIRT1). In sepsis, this balance is disrupted, with excessive M1 activation driving inflammation and insufficient M2 activity delaying tissue repair.

However, the balance between M1 and M2 macrophages is often disrupted during sepsis, and insufficient M2 activity may contribute to persistent inflammation and delayed tissue repair (**Figure 2**) (15). Moreover, emerging evidence suggests that a phenomenon termed “delayed sustained M1 activation” can occur during the later stages of sepsis, in which macrophages maintain a proinflammatory phenotype even after the inflammatory environment has subsided. This process may be associated with persistent HIF-1α expression induced by tissue hypoxia and impaired metabolic reprogramming (34).

#### 2.2.2 Enhanced neutrophil activity and neutrophil extracellular trap formation (NETosis)

**Figure 3:**
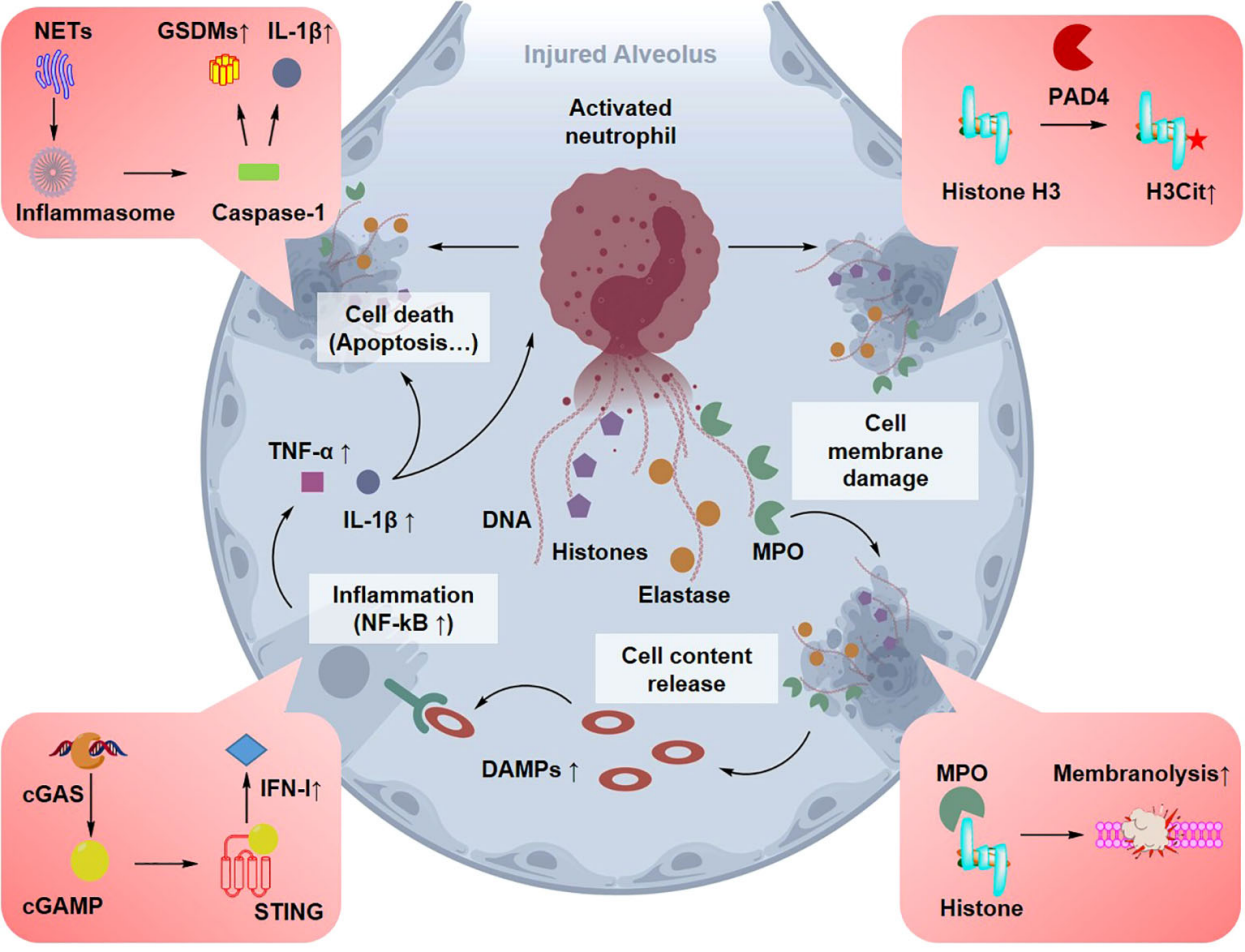
Mechanisms of NETosis and their destructive effects on the alveoli. Activated neutrophils release NETs composed of DNA, histones (e.g., CitH3), and enzymes (e.g., MPO). While intended for pathogen clearance, excessive NETs in the alveolar space induce direct cytotoxicity to epithelial cells, trigger thrombosis via the STING pathway, and release DAMPs that amplify the inflammatory cycle.

Neutrophils are excessively activated in sepsis-associated ALI, leading to extensive formation of NETs, a process referred to as NETosis (16). NETs are web-like structures composed of decondensed chromatin fibers decorated with antimicrobial proteins such as MPO, neutrophil elastase, and histones. During NETosis, nuclear chromatin is released into the extracellular space to trap and neutralize invading pathogens. Although NET formation contributes to host defense, excessive or dysregulated NETosis can cause substantial tissue injury in the lung. In sepsis-associated ALI, excessive NET accumulation disrupts alveolar epithelial and endothelial barriers, promotes inflammatory amplification, and contributes to immunothrombosis and microvascular injury, thereby aggravating pulmonary edema and respiratory dysfunction (40). Key NET components, including MPO and histones, can directly damage cellular membranes and induce apoptosis or necrosis, leading to the release of damage-associated molecular patterns (DAMPs) and the amplification of inflammatory signaling (**Figure 3**) (41). DAMPs, such as histones, cell-free DNA, and HMGB1, can activate receptors including TLR9 and RAGE, sustaining immune cell activation and driving endothelial cell apoptosis and barrier dysfunction (42). In addition, NETs engage in bidirectional regulatory interactions with immune cell populations, such as regulatory T cells (Tregs) and DCs, thereby contributing to the disruption of the alveolar microenvironment and the exacerbation of tissue injury (43).

#### 2.2.3 DC dysfunction and impaired antigen presentation (DC exhaustion)

**Figure 4:**
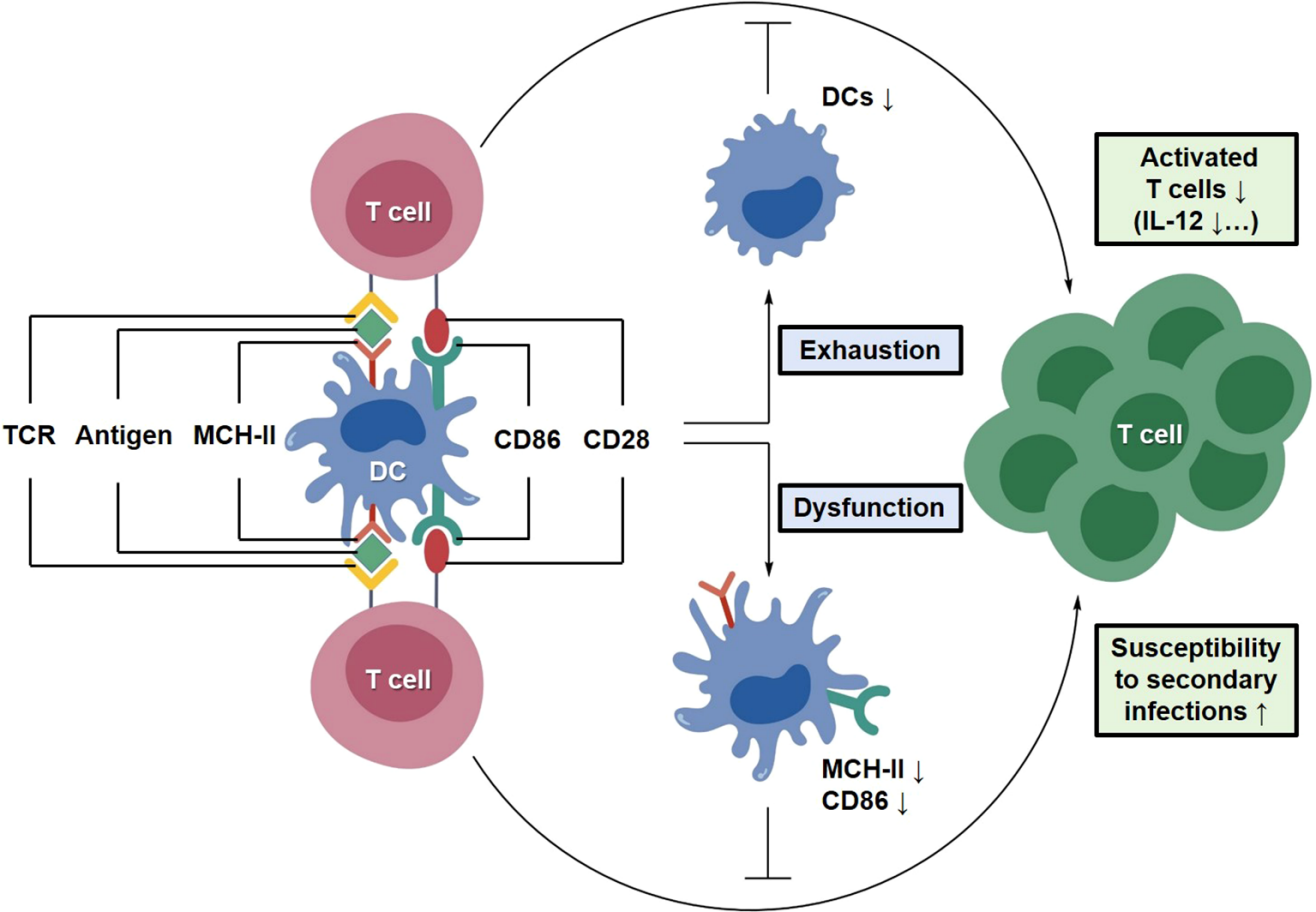
Mechanisms underlying DC exhaustion and adaptive immune inactivation. During sepsis progression, DCs undergo functional exhaustion, characterized by reduced numbers, downregulation of antigen-presenting molecules (MHC-II, CD86), and increased apoptosis. This leads to impaired T cell activation and a state of immunoparalysis, increasing susceptibility to secondary infections.

During sepsis, DC numbers decline markedly and their functional capacity becomes compromised, leading to impaired antigen presentation (44). This reduction has been linked to defects in DC precursor differentiation, accompanied by decreased expression of major histocompatibility complex class II (MHC-II) and costimulatory molecules such as CD86 (45). Concurrently, inflammation-driven programmed cell death processes, including apoptosis and ferroptosis, further exacerbate the depletion of DC populations (44). DC exhaustion weakens adaptive immune responses and increases susceptibility to secondary infections, thereby promoting the development of sepsis-associated immunoparalysis (46). In addition, impaired DC function limits the activation and differentiation of T cells, further aggravating immune imbalance (**Figure 4**). Experimental evidence indicates that the capacity of DCs to activate CD4^+^ and CD8^+^ T cells is significantly diminished, directly resulting in a functional “breakdown” of the adaptive immune cascade (46). Consequently, DC dysfunction is considered a key mechanistic link between the early hyperinflammatory phase and the subsequent immunosuppressive state observed in sepsis (47).

#### 2.2.4 Aberrant activation of key signaling pathways: molecular basis of inflammatory amplification

In sepsis-associated ALI, multiple signaling pathways are aberrantly activated and collectively drive immunopathological responses (48). Among these, the TLR4/NF-κB pathway recognizes PAMPs, such as bacterial endotoxins, thereby activating downstream transcription factors that induce the release of proinflammatory cytokines, including TNF-α, IL-6, and IL-1β. This pathway initiates systemic inflammatory responses and represents one of the central signaling axes underlying the cytokine storm (49). Excessive TLR4 signaling also promotes upregulation of the immunosuppressive molecule PD-L1, thereby contributing to immune imbalance (50). Further studies indicate that the TLR4–NF-κB axis cooperatively activates signal transducer and activator of transcription 3 (STAT3) and interferon regulatory factor 1 (IRF1), which enhances PD-L1 transcription and promotes immune tolerance in antigen-presenting cells, suppresses T cell activation, and contributes to persistent immunosuppression during the late stages of sepsis (51).

The NLRP3 inflammasome, a critical intracellular inflammatory sensor, responds to the accumulation of ROS and DAMPs, leading to caspase-1 activation, IL-1β and IL-18 maturation and secretion, and induction of pyroptosis, thereby amplifying local tissue injury (25). Recent evidence indicates that METTL14 stabilizes NLRP3 mRNA via an m^6^A-dependent mechanism, increasing NLRP3 expression and promoting inflammasome activation and pyroptosis, which aggravates lung injury (52). Conversely, heat shock factor 1 (HSF1) negatively regulates NLRP3 inflammasome activity by suppressing NF-κB signaling and promoting NLRP3 ubiquitination, thereby exerting protective effects in experimental models of sepsis-associated ALI (53). In addition to NLRP3, other inflammasome-related pathways may also contribute to the inflammatory landscape of acute lung injury. Recent evidence indicates that TRIM29 can regulate NLRP6 inflammasome activation (54), while NLRP6 has also been implicated in the control of acute lung inflammation (55). Given the growing evidence that EVs can modulate inflammasome activity through the transfer of regulatory miRNAs, proteins, and other bioactive cargoes, it is plausible that EVs may alleviate acute lung injury, at least in part, by regulating TRIM29 expression and thereby influencing TRIM29-mediated inflammasome activation. However, direct evidence linking EVs to the TRIM29–NLRP6 axis in sepsis-associated ALI is currently lacking, and this potential mechanism should therefore be regarded as an emerging but still speculative direction for further investigation.

Recent studies have also highlighted the importance of the cyclic GMP–AMP synthase–stimulator of interferon genes (cGAS–STING) pathway, an intracellular DNA-sensing system that becomes highly activated in sepsis-associated ALI. Bacterial or mitochondrial DNA released during cellular injury can activate STING-mediated type I interferon signaling, which promotes alveolar epithelial cell injury and amplifies inflammatory responses in the lung (56–58).

Beyond these classical pathways, additional signaling axes contribute to inflammatory amplification and immunometabolic dysregulation in sepsis-associated ALI. The MAPK pathways, including p38, ERK, and JNK, promote activation of transcription factors such as AP-1, thereby enhancing inflammatory responses in neutrophils and macrophages. Evidence indicates that eriocitrin alleviates sepsis-induced ALI by modulating glycolysis through the MKP1/MAPK signaling pathway (56). The JAK/STAT axis exerts a dual regulatory role by balancing proinflammatory signaling (e.g., STAT1/3 activation) and immunosuppressive processes (e.g., IL-10 and PD-L1 expression). Notably, sufentanil has been shown to ameliorate lung injury in sepsis mouse models by inhibiting the JAK2-STAT3 signaling pathway, providing evidence for the pivotal involvement of this axis in inflammatory regulation (59). In addition, the mammalian target of rapamycin (mTOR)/AMP-activated protein kinase (AMPK) axis regulates cellular metabolism, autophagy, and pyroptosis, thereby influencing alveolar epithelial and immune cell homeostasis. Activation of AMPK and inhibition of mTOR signaling, such as through ketamine treatment, enhance autophagy and attenuate apoptosis in alveolar epithelial cells, ultimately mitigating lung injury (60).

**Figure 5:**
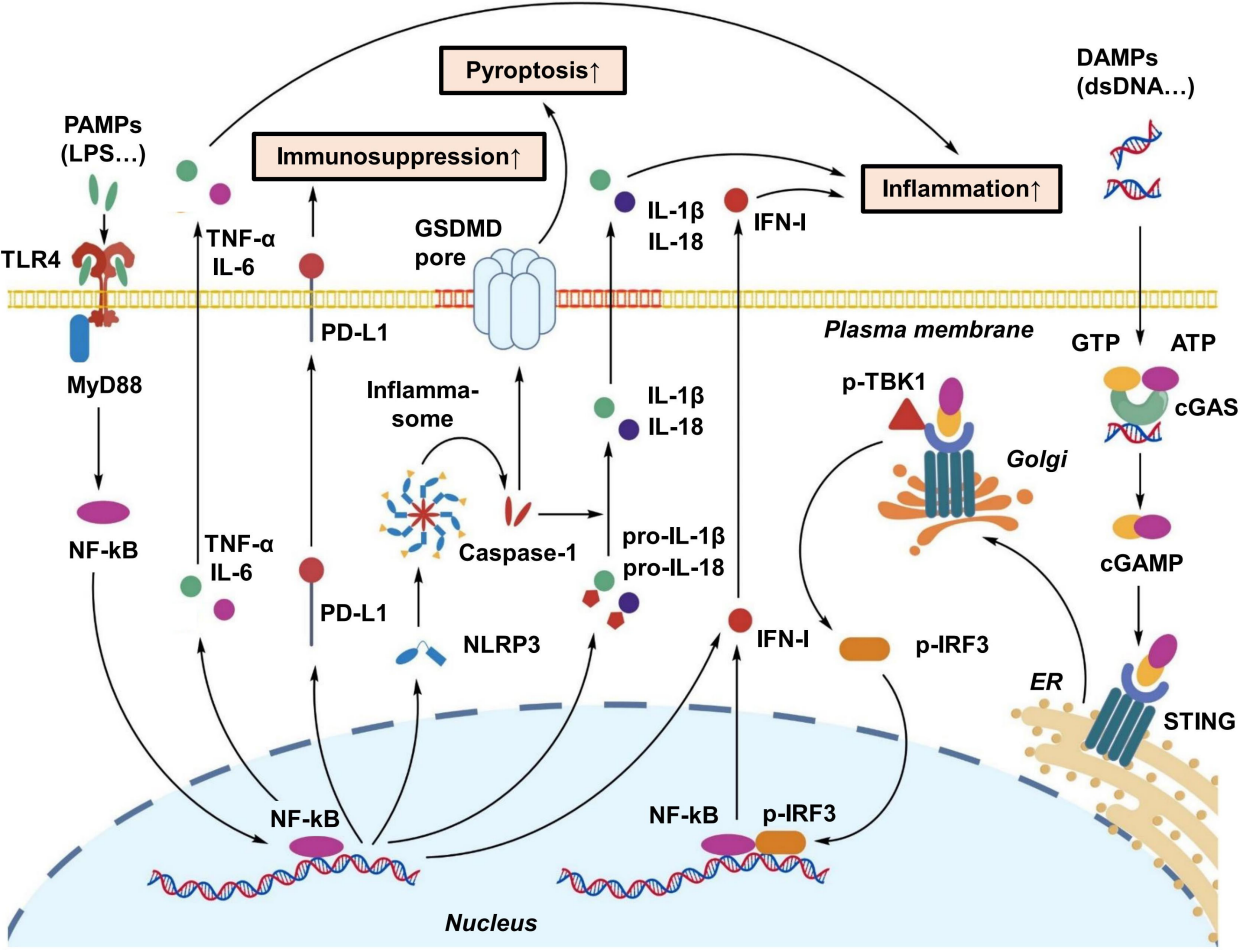
Activation network of key innate immune signaling pathways (TLR4/NLRP3/cGAS-STING). This diagram illustrates the crosstalk between major inflammatory pathways. TLR4 recognizes PAMPs to activate NF-κB; the NLRP3 inflammasome responds to ROS/DAMPs to trigger pyroptosis and IL-1β release; and the cGAS-STING pathway senses cytosolic DNA to induce type I interferons. These pathways collectively drive the immunopathology of ALI.

These signaling pathways exhibit distinct spatiotemporal expression patterns across different immune cell types, including macrophages, neutrophils, and DCs, and interact to form a multilayered regulatory network that drives the progression of sepsis-associated ALI. Subsequent sections discuss how EVs may influence these signaling axes to modulate immune responses and promote tissue protection (**Figure 5**). A detailed summary of major signaling pathways implicated in sepsis-associated ALI and their regulatory mechanisms is provided in **Table 1** (40, 52, 53, 56, 59–62).

**Table 1:** Recent advances and mechanistic overview of key signaling pathways involved in sepsis-associated acute lung injury over the past three years.

Pathway/mechanism	Key regulators/targets	Summary of findings and mechanistic insights
mTOR/AMPK (60)	AMPK, mTOR, ketamine	Ketamine activated AMPK and inhibited the mTOR pathway, thereby enhancing autophagy, reducing alveolar epithelial cell apoptosis, and alleviating lung injury.
JAK/STAT (59)	JAK2, STAT3, sufentanil	Sufentanil improved lung injury in septic mouse models by inhibiting the JAK2–STAT3 signaling pathway, providing evidence for the critical role of this axis in inflammatory regulation.
MAPK (56)	MKP1, p38, ERK, JNK, eriocitrin	Eriocitrin attenuated sepsis-induced acute lung injury by regulating MKP1/MAPK pathway–mediated glycolysis.
NLRP3 inflammasome (52)	METTL14, NLRP3, m^6^A modification	METTL14 stabilized NLRP3 mRNA through m^6^A modification, enhancing NLRP3 expression and inflammasome activation, thereby promoting pyroptosis and exacerbating lung tissue injury.
NLRP3 inflammasome (53)	HSF1, NF-κB, NLRP3	Heat shock factor 1 (HSF1) negatively regulated NLRP3 inflammasome activity by suppressing NF-κB activation and promoting NLRP3 ubiquitination, exerting a protective effect in sepsis-associated ALI models.
cGAS–STING pathway (40)	cGAS, STING, IFN-I	As an intracellular DNA-sensing mechanism, the cGAS–STING pathway was highly activated in sepsis-associated ALI; cytosolic bacterial or mitochondrial DNA released after cell necrosis triggered STING-mediated type I interferon signaling, thereby aggravating alveolar epithelial injury and immune responses.
cGAS–STING pathway (61)	cGAS, STING, IFN-I	The cGAS–STING pathway functioned as a cytosolic DNA sensor and was strongly activated in sepsis-associated ALI, particularly in response to bacterial or mitochondrial DNA released following cellular necrosis, leading to enhanced STING-dependent IFN-I signaling and pulmonary injury.
TLR4/NF-κB (62)	TLR4, NF-κB, PD-L1, STAT3, IRF1	Excessive TLR4 activation induced upregulation of the immunosuppressive molecule PD-L1, thereby exacerbating immune imbalance. Further studies demonstrated that the TLR4–NF-κB pathway synergistically activated STAT3 and IRF1 to enhance PD-L1 transcription, resulting in antigen-presenting cell tolerance, suppression of T-cell activation, and sustained immunoparalysis during late-stage sepsis.

## 3 EVs and biomimetic nanocarriers: biological foundations and engineering strategies

### 3.1 Native EVs and their innate immune regulatory properties

EVs, particularly exosomes, are nanoscale membrane-bound vesicles with diameters of approximately 30–150 nm that are secreted by various cell types, including MSCs, macrophages, and DCs (63). The biological functions of EVs largely depend on the characteristics of their parent cells. For example, mesenchymal stem cell-derived extracellular vesicles (MSC-EVs) exhibit considerable potential in tissue repair and immune regulation and have been widely investigated in inflammatory diseases (64). Macrophage-derived EVs participate in the regulation of inflammatory responses and innate immune activity (15), whereas DC-derived EVs possess antigen-presenting properties that make them promising candidates for vaccine development and immunotherapeutic applications (65).

EVs carry diverse bioactive cargoes, including miRNAs, long non-coding RNAs (lncRNAs), and proteins. Among these, miRNAs are key regulators of gene expression and immune responses. Multiple studies have demonstrated that MSC-EVs are enriched in immunomodulatory miRNAs, such as miR-223 and miR-146a, which may influence innate immune activity and inflammatory signaling (66, 67). In addition to miRNAs, lncRNAs, and inflammation-related proteins are also thought to contribute to EV-mediated immunoregulation, although their precise mechanisms remain incompletely understood.

The EV membrane surface is also enriched with proteins involved in immune recognition and intercellular communication. MHC-II, a central molecule in antigen presentation, is abundantly expressed on EVs derived from DCs and contributes to CD4^+^ T cell activation and adaptive immune responses (68). HSP70, a classical stress-response protein, plays roles in EV biogenesis and secretion and can activate TLR2/TLR4 signaling pathways to enhance innate immune responses (69). In addition, adhesion molecules such as ICAM-1 facilitate the binding and uptake of EVs by target cells, including T cells and endothelial cells, thereby improving tissue-specific targeting efficiency (70). In addition, costimulatory molecules such as CD86 and CD40 are commonly expressed on EVs derived from DCs and macrophages and participate in T cell activation and immunological synapse formation (71). Collectively, these molecular components contribute to EV-mediated intercellular communication and immune modulation.

**Figure 6:**
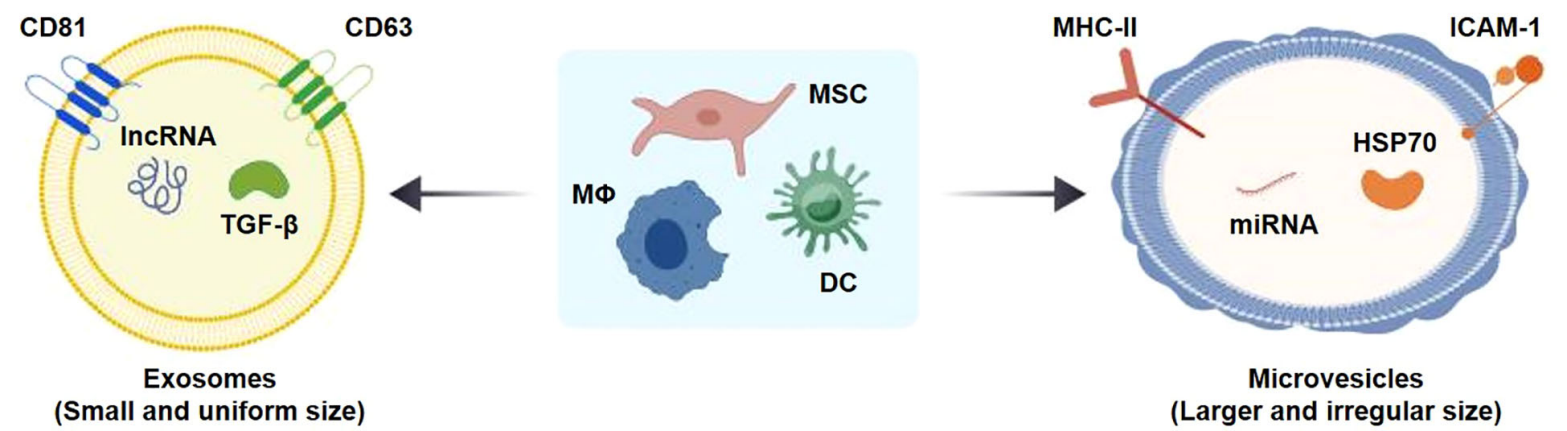
Structural features of EVs and their immunoregulatory miRNA and protein cargoes. EVs are lipid bilayer-enclosed nanovesicles carrying bioactive molecules. Key cargoes include immunomodulatory miRNAs (e.g., miR-223, miR-146a) and surface proteins (e.g., MHC-II, CD47, PD-L1) that facilitate intercellular communication and immune regulation.

Multiple studies have demonstrated that native EVs exert immunomodulatory and tissue-protective effects in models of ALI. In particular, MSC-EVs have been reported to attenuate inflammatory responses by influencing macrophage polarization through miRNA-mediated mechanisms (72). In addition, EVs reduce alveolar epithelial cell damage by inhibiting the formation of NETs (73). These findings highlight the potential of EV-based approaches for the treatment of ALI, although further mechanistic studies are required to clarify their precise regulatory roles (**Figure 6**).

### 3.2 Bioengineering of EV-based biomimetic nanocarriers

#### 3.2.1 Construction strategies and preparation techniques of biomimetic carriers

EV membrane-coated nanoparticles represent a biomimetic strategy that integrates synthetic nanoparticles with natural EV membranes. Encapsulation of nanoparticles within EV membranes confers EV-like biocompatibility and targeting capability (74). For example, drug-loaded nanoparticles coated with EV membranes can facilitate targeted delivery to specific tissues or cell types while reducing nonspecific distribution and potential adverse effects (75). In addition, EV membrane coating improves nanoparticle stability and prolongs systemic circulation, thereby enhancing therapeutic performance (74).

Membrane fusion-based approaches involve integrating cell membranes from different sources with EV membranes to generate multifunctional biomimetic nanovesicles. This strategy has demonstrated advantages in targeted delivery and immune modulation. More recently, multi-cell membrane fusion strategies have been developed, in which membranes derived from different cell types, such as immune cells, tumor cells, or endothelial cells, are combined to achieve synergistic functions including enhanced targeting, immune evasion, and improved biological barrier penetration. In complex disease models such as cancer and inflammatory disorders, biomimetic nanocarriers constructed through multi-membrane fusion have shown promising therapeutic potential (76, 77). By integrating complementary membrane properties, these systems enable simultaneous engagement of multiple biological targets. For example, fusion of tumor cell membranes with EV membranes can produce nanocarriers with enhanced tumor-targeting capacity for anticancer drug delivery (76). In addition, membrane fusion-derived vesicles have been explored in vaccine development and immunotherapy, highlighting their broad translational potential (78).

The fabrication of EV-based biomimetic nanocarriers employs various engineering techniques, including microfluidic technologies and electrofusion. Surface ligand modification has also emerged as an effective strategy for improving targeting specificity. Functionalization of EV surfaces with peptides, antibodies, or other ligands enhances recognition of target cells and improves tissue penetration, thereby increasing therapeutic selectivity (79, 80). Microfluidic platforms enable controllable and scalable fabrication by regulating membrane fusion efficiency, particle size distribution, and surface functionalization, making them increasingly important in biopharmaceutical manufacturing (81, 82). Compared with conventional methods such as ultrasonication and extrusion, microfluidic chips offer superior reproducibility, tunability, and industrial translation potential (81, 83). In contrast, electrofusion induces membrane fusion between EVs and synthetic nanocarriers through external electric fields, generating stable hybrid vesicles with high cargo encapsulation efficiency while preserving membrane proteins (79).

**Figure 7:**
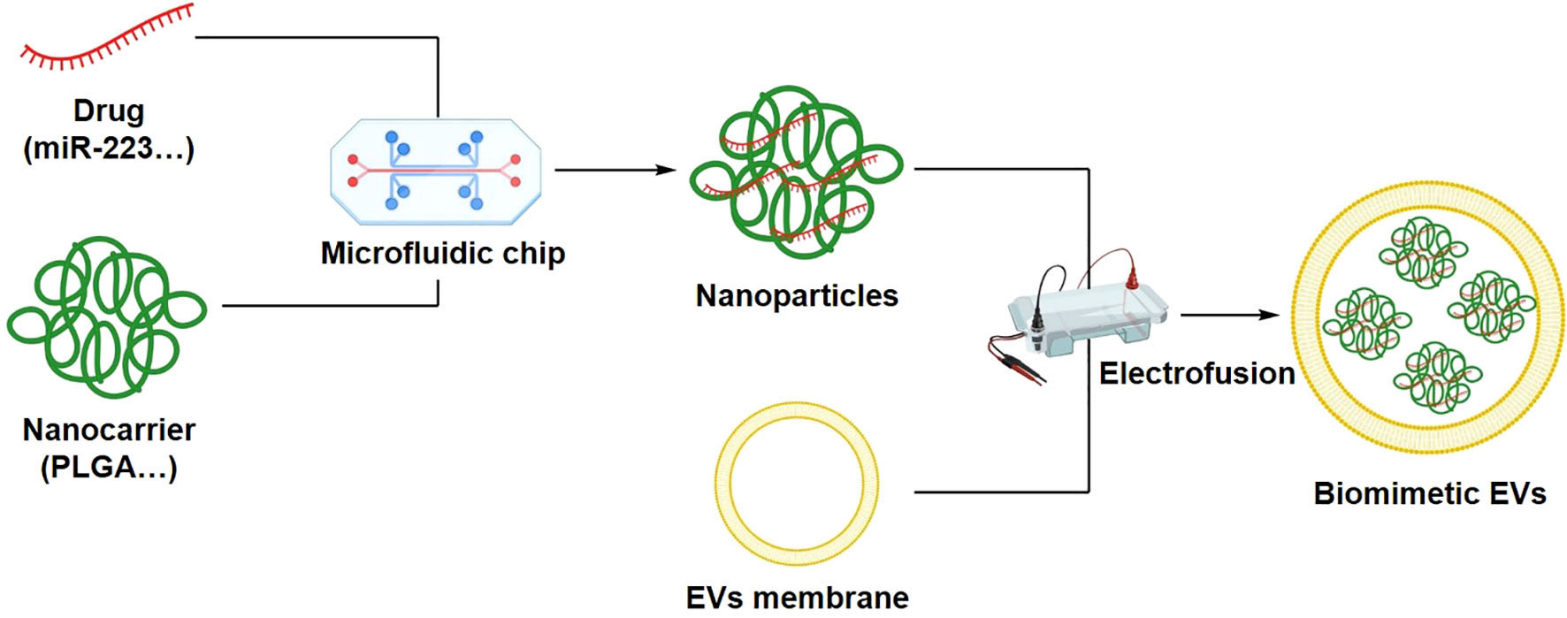
Engineering workflow for the construction of EV-based biomimetic nanosystems. A step-by-step overview of preparing biomimetic carriers, including EV isolation, drug loading (via electroporation or incubation), and surface engineering techniques such as membrane fusion, coating synthetic nanoparticles, and ligand modification to enhance stability and targeting.

To further enhance functional versatility, advanced engineering strategies such as multi-cell membrane fusion and surface ligand modification can be incorporated into the fabrication process. Multi-membrane fusion integrates biological properties from different cell types, endowing nanocarriers with enhanced targeting capability and immunoregulatory potential (76, 84, 85). Concurrently, ligand modification improves target cell recognition and tissue penetration by decorating EV surfaces with specific ligands, thereby further increasing therapeutic selectivity (86) (**Figure 7**).

#### 3.2.2 Targeting properties and immunological advantages of biomimetic systems

EV-based biomimetic nanocarriers preserve key membrane proteins from their parental cells, enabling partial retention of natural biological functions. This property contributes to a characteristic “immune stealth” profile that can reduce rapid clearance and improve circulation stability *in vivo*, which may be advantageous in inflammatory conditions such as sepsis-associated ALI (74). Experimental evidence suggests that this effect is partly mediated by CD47 expressed on EV membranes, which activates signal regulatory protein α (SIRPα)–dependent “don’t eat me” signaling and suppresses macrophage-mediated phagocytosis, thereby prolonging systemic circulation and reducing nonspecific clearance (87). Compared with conventional synthetic carriers, including poly(lactic-co-glycolic acid) (PLGA) nanoparticles, liposomes, and polyethylenimine (PEI)-based systems, EV-derived biomimetic carriers tend to exhibit improved biocompatibility and more controlled interactions with immune cells (74). Strategies aimed at enhancing CD47 expression or incorporating immune-regulatory ligands, such as TSP-1–mimetic peptides or SIRPα-binding motifs, have been explored to further strengthen immune evasion. In addition, incorporation of complement regulatory proteins such as CD55 and CD59 may help attenuate complement activation and innate immune recognition (74).

**Figure 8:**
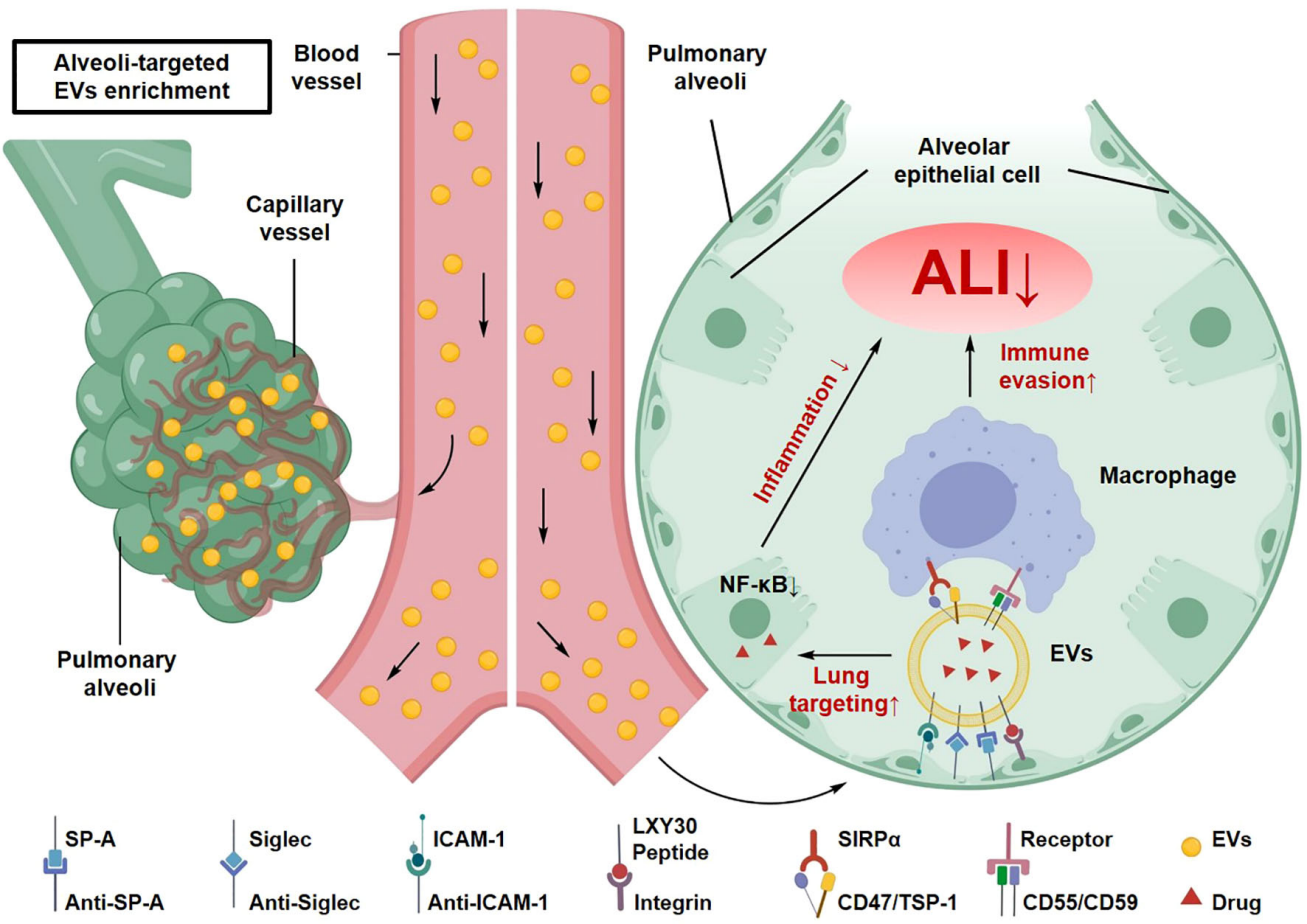
Schematic illustration of active pulmonary targeting mechanisms of EV-based biomimetic systems. Engineered EVs can be modified with surface ligands (e.g., antibodies, peptides) that specifically recognize pulmonary receptors like SP-A, Siglecs, or ICAM-1. This active targeting strategy, combined with passive accumulation, enhances the delivery of therapeutic cargoes to injured lung tissue.

Beyond immune evasion, biomimetic nanocarriers may also benefit from the anatomical characteristics of the lung. The pulmonary microvasculature features a dense capillary network and relatively high endothelial permeability, which can facilitate passive accumulation of nanoparticles. Engineering strategies that incorporate ligands or antibodies targeting pulmonary receptors, such as surfactant protein A (SP-A), sialic acid–binding immunoglobulin-like lectins (Siglecs), or ICAM-1, have therefore been investigated to enhance active lung targeting (88). For example, stem cell–derived small extracellular vesicles (sEVs) enriched with miR-21-5p have been reported to alleviate lipopolysaccharide (LPS)-induced ALI through suppression of the inflammation-associated target PCSK6 (89). In another study, biomimetic nanoparticles constructed using MSC-EV membranes and decorated with alveolar-targeting peptides demonstrated enhanced accumulation in the lungs of ALI mice. Delivery of miR-21 through these systems reduced NF-κB signaling activity, attenuated pulmonary inflammation, and improved oxygenation indices, illustrating the potential of biomimetic EV-based platforms for immune modulation in ALI (**Figure 8**) (89).

## 4 EV-based strategies for innate immune remodeling

### 4.1 Macrophage reprogramming: transition from M1 to M2 phenotypes

**Figure 9:**
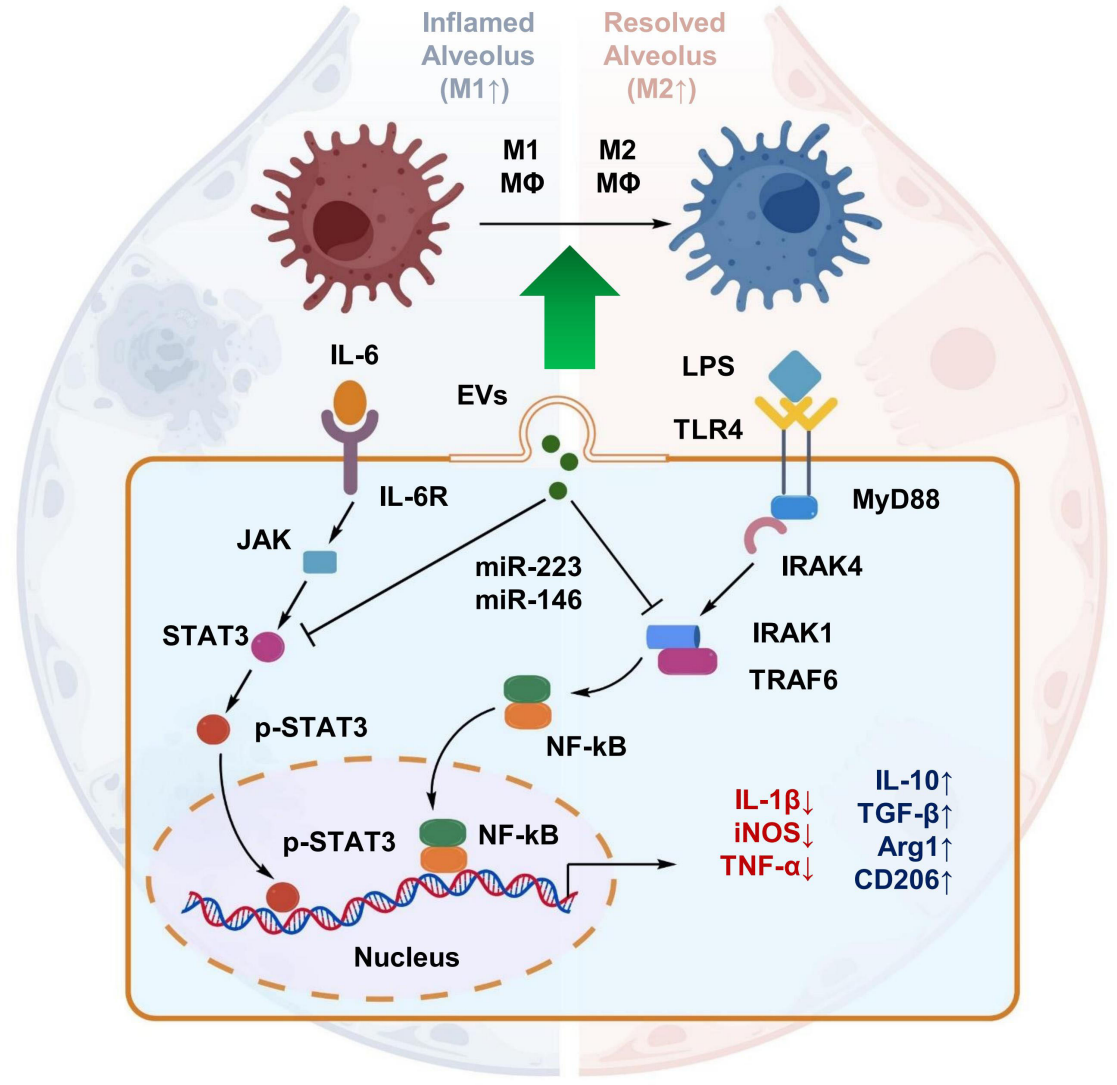
Mechanisms of EV-mediated macrophage polarization from the M1 to the M2 phenotype. EVs, particularly those derived from MSCs, deliver miRNAs (e.g., miR-223) that suppress pro-inflammatory signaling (STAT3, TRAF6). This reprograms macrophages from the damaging M1 state to the reparative M2 state, promoting the resolution of inflammation.

Macrophage polarization status plays an important role in the immunopathology of sepsis-associated ALI (16). During sepsis, macrophages can dynamically transition between proinflammatory M1 and anti-inflammatory M2 phenotypes, and disruption of this balance contributes to excessive inflammation and impaired tissue repair in the lung. M1 macrophages exhibit pronounced proinflammatory characteristics and secrete mediators such as IL-1β, TNF-α, and inducible nitric oxide synthase (iNOS), thereby activating NF-κB signaling and amplifying inflammatory responses. In contrast, M2 macrophages release anti-inflammatory cytokines, including IL-10 and TGF-β, and promote tissue repair and resolution of inflammation (90). Recent evidence indicates that EV membrane proteins can influence macrophage polarization outcomes. For example, VCAM-1 expressed on certain EVs can bind α4β1 integrins on monocytes, promoting their recrutment and polarization toward an M1 phenotype (91). In contrast, MSC-EVs are enriched in immunomodulatory miRNAs, including miR-223 and miR-146a, which may facilitate macrophage repolarization toward the M2 phenotype by suppressing signaling pathways such as STAT3, IRAK1, and TRAF6 (66, 92). For instance, in a LPS-induced ALI model, increased miR-223 expression was associated with reduced levels of M1-related markers and increased expression of M2 markers such as Arg1 and CD206, highlighting the role of EV-derived miRNAs in macrophage polarization (**Figure 9**) (66).

Beyond miRNA delivery, engineered EVs have also been explored as carriers for anti-inflammatory cytokines such as IL-4 and IL-10, which can promote macrophage reprogramming toward an anti-inflammatory phenotype. The regulatory effects of EVs on macrophage polarization appear to depend strongly on their cellular origin. For example, EVs derived from endothelial progenitor cells preferentially promote M2 polarization, whereas endothelial cell-derived EVs have been reported to induce M1 activation through molecules such as S100A8/A9, thereby exacerbating pulmonary inflammation (93, 94). Mechanistically, IL-4 activates the STAT6 signaling pathway to initiate the M2 transcriptional program, while IL-10 suppresses NF-κB signaling and attenuates inflammatory amplification (90). Several studies have further demonstrated that hybrid delivery systems combining liposomes and EV membranes can enhance pulmonary delivery of anti-inflammatory cytokines. For example, liposome–EV hybrid carriers loaded with IL-10 significantly increased the proportion of M2-polarized alveolar macrophages in cecal ligation and puncture (CLP) models (95). In addition, MSC-EVs naturally carry M2-promoting factors, including TGF-β and miR-21, which may contribute to modulation of pulmonary macrophage phenotypes and improvements in pathological indicators including pulmonary edema and inflammatory scores (96).

Potential involvement of the TRIM29–PERK axis in EV-mediated macrophage reprogramming should also be considered. TRIM29 has been identified as a regulator of innate immune responses in alveolar macrophages during bacteria-induced sepsis-associated lung injury, and Trim29 deficiency was reported to enhance antibacterial responses and protect mice from sepsis-induced lung injury. In addition, recent evidence showed that TRIM29 promotes PERK SUMOylation and stabilizes PERK, whereas PERK signaling has been implicated in driving immunosuppressive M2-like macrophage polarization. Given that EVs can reshape macrophage polarization through delivery of immunoregulatory miRNAs, proteins, and cytokines, it is plausible that part of their protective effect in acute lung injury may involve modulation of TRIM29 expression and/or the TRIM29–PERK signaling axis, thereby limiting maladaptive macrophage reprogramming. However, direct evidence linking EVs to TRIM29-dependent PERK regulation in sepsis-associated ALI is still lacking, and this mechanism should currently be regarded as a promising but speculative direction for future investigation (97–99).

### 4.2 Neutrophil-mediated tissue injury and regulation of NETs

NETs play an essential role in pathogen clearance; however, excessive NETosis constitutes a critical pathological process in ALI (40). NETs consist of extracellular DNA decorated with antimicrobial proteins such as MPO and histones, particularly citrullinated histone H3 (CitH3), which exhibit strong cytotoxic effects on alveolar epithelial cells (100).

Emerging evidence indicates that EVs may participate in the regulation of NETosis. For instance, the cleaved fragment of gasdermin D (GSDMD-N) can be transported into EVs via mitochondrial pathways, thereby promoting NET release and suggesting a potential role for DC-derived EVs in NET regulation (101). In addition, activation of the STING signaling pathway by NETs has been identified as a central driver of immunothrombosis and exacerbated lung injury, highlighting the potential of EV-mediated modulation of the STING pathway as a therapeutic strategy (28). In multiple ALI animal models, NET accumulation has been positively correlated with protein levels in alveolar exudates and inflammatory cytokine concentrations. In LPS-induced ALI models, extensive MPO- and CitH3-positive staining is observed within alveolar spaces and is closely associated with the severity of ARDS (40).

**Figure 10:**
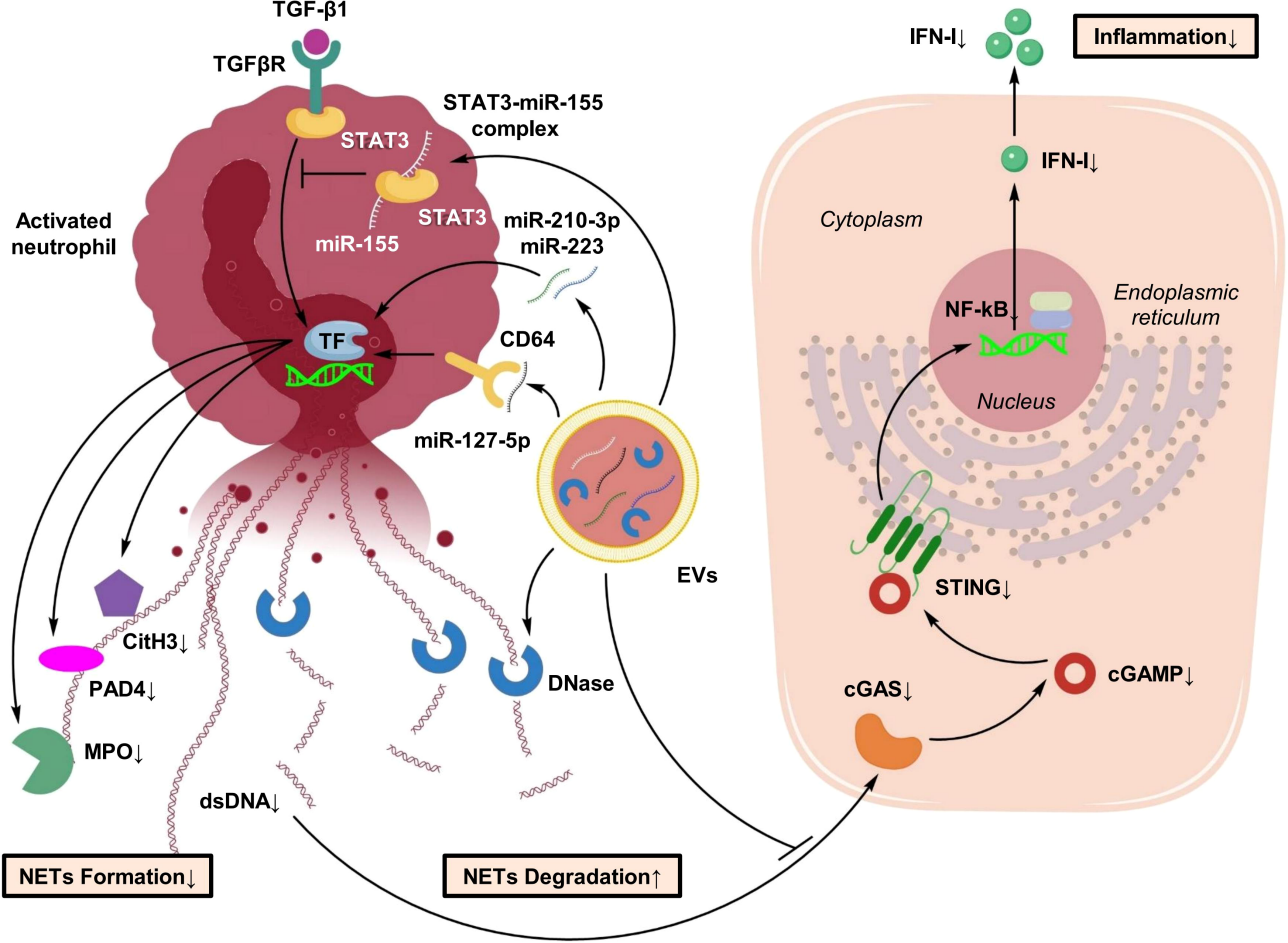
Dual pathways by which EVs regulate NETosis and degradation. EVs modulate neutrophil activity by inhibiting NET formation (e.g., via miR-127-5p targeting CD64) and by promoting the degradation or clearance of existing NETs. This dual action helps mitigate NET-induced alveolar injury and immunothrombosis.

Current evidence suggests that EVs may modulate NETs through two principal mechanisms: inhibition of NETosis and promotion of NET degradation (102). Regarding NET inhibition, MSC-EVs are enriched with regulatory miRNAs that influence neutrophil activation. For example, miR-127-5p has been shown to inhibit NET release by targeting the Fcγ receptor CD64 on neutrophils, thereby reducing lung tissue injury (103). MSC-EVs have also been reported to attenuate NETosis by suppressing ROS-dependent signaling and inhibiting ferroptosis, resulting in enhanced tissue protection (104). In addition, EV-mediated delivery of miR-223 has been shown to suppress NET formation and improve lung injury outcomes in models of sepsis-associated ALI (**Figure 10**) (103).

### 4.3 Restoration of DC function and antigen presentation

During the late stages of sepsis or the progression of ALI, DCs undergo a marked reduction in number and functional exhaustion, which contributes to immunoparalysis and increased susceptibility to secondary infections (44). In lung tissue, impaired antigen-presenting capacity of DCs, characterized by decreased expression of MHC-II and CD86, results in defective initial T cell activation and creates a critical window of immune vulnerability (45). Clinical analyses further indicate that even during early recovery, sepsis survivors often exhibit persistently reduced DC counts and impaired IL-12 production compared with healthy individuals, which correlates with prolonged hospitalization and increased mortality (45).

**Figure 11:**
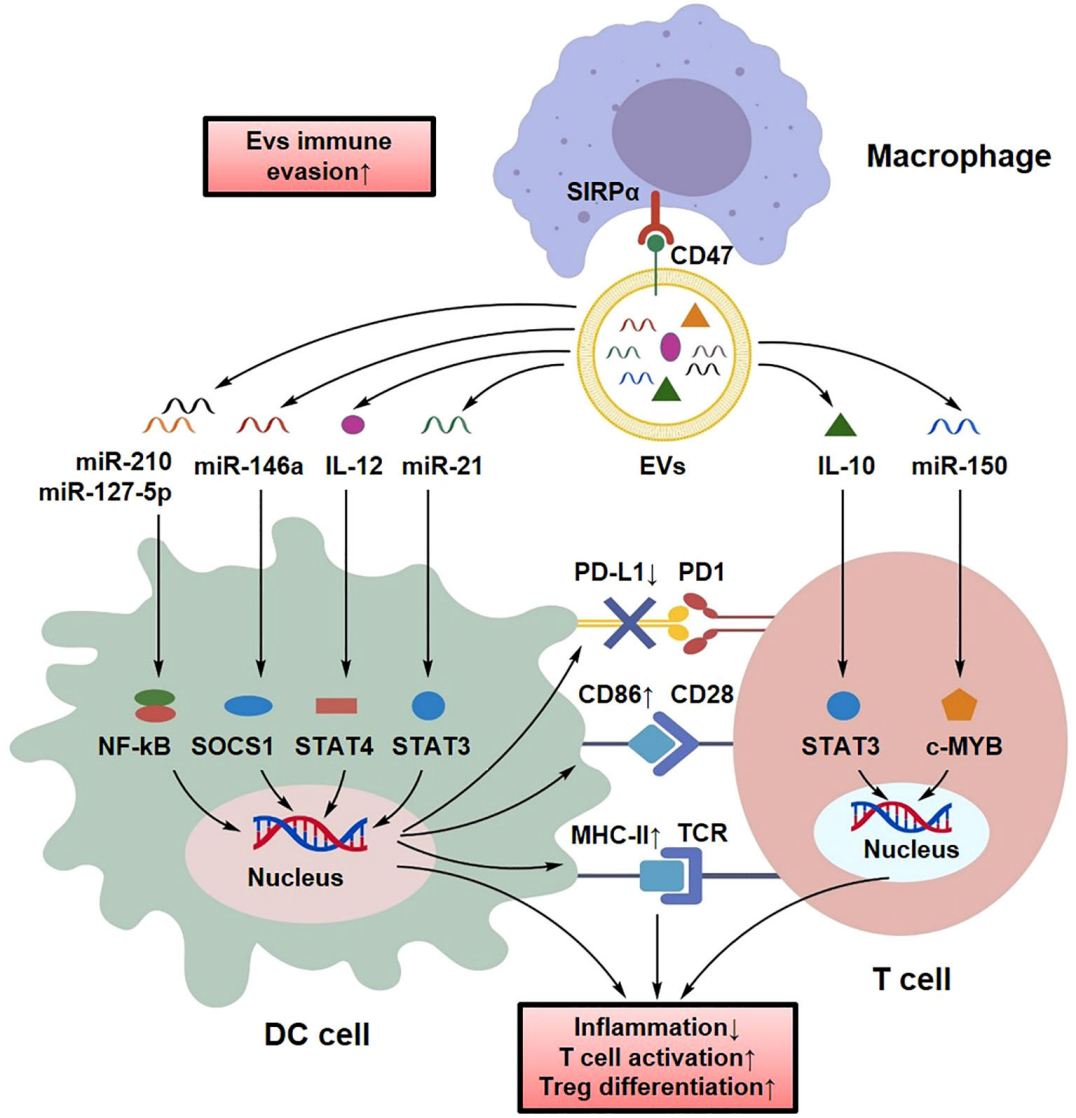
EV-mediated regulation of DC functional restoration and antigen presentation networks. Engineered EVs can restore dendritic cell function by delivering specific miRNAs or antigens. This enhances the expression of costimulatory molecules (CD80/CD86) and MHC-II, thereby reinvigorating antigen presentation and restoring adaptive immune responses.

EVs have emerged as potential platforms for modulating DC function through the delivery of regulatory RNA and protein cargoes (105). For example, miR-146a carried by MSC-EVs has been reported to suppress DC maturation and inflammatory antigen presentation (106). In addition, EV-enriched miR-21 has been shown to enhance DC antigen-presenting function via the STAT3 pathway and promote Treg induction, thereby contributing to immune tolerance (107). In addition, cytokine-loaded EV systems have been reported to enhance DC activation by increasing the expression of antigen-presenting molecules such as MHC-II and CD86, thereby promoting CD4^+^ T cell responses in experimental inflammatory models (95). Beyond direct regulation of DCs, EV-mediated modulation of other immune cells may also indirectly influence DC-related immune responses. For instance, in models of sepsis-associated ALI, bone marrow MSC-derived exosomes carrying miR-127-5p have been shown to target the neutrophil Fcγ receptor CD64, thereby suppressing NETosis and alleviating pulmonary inflammation (103). Single-cell immune atlas analyses have further identified the emergence of immunoregulatory DC subsets during sepsis progression, which promote naïve T cell activation and facilitate Treg differentiation (107). In addition, CD47 expressed on EV surfaces can activate “don’t eat me” signaling, reducing macrophage-mediated clearance and prolonging systemic circulation, which may enhance EV accumulation in DC-enriched pulmonary regions (**Figure 11**) (108).

### 4.4 EV-targeted interventions of innate immune signaling pathways

The TLR4 pathway, the NLRP3 inflammasome, and the cGAS-STING signaling axis represent key inflammatory hubs in sepsis-associated ALI. Due to their ability to transport regulatory RNAs and proteins, EVs have emerged as promising platforms for modulating these signaling pathways (61, 109). MSC-EVs are enriched in miR-21 and miR-223, which target TLR4 and MyD88, respectively, thereby suppressing downstream NF-κB signaling and reducing the production of proinflammatory cytokines including IL-6 and IL-1β (110). Emerging evidence further suggests that EVs may regulate inflammatory signaling by influencing the expression of TLR4 adaptor proteins, including TIRAP and TRIF, providing potential molecular targets for EV-based therapeutic design (105). Another study demonstrated that human umbilical cord-derived mesenchymal stem cell (hucMSC) EVs delivering miR-146a-5p attenuated inflammatory responses in ALI, highlighting the anti-inflammatory potential of miR-146a-loaded EVs (92). Histone deacetylase 3 (HDAC3) has been identified as an upstream regulator that exacerbates LPS-induced ALI by activating the cGAS–STING signaling pathway and promoting macrophage pyroptosis. HDAC3-mediated regulation of histone acetylation further enhances STING pathway activation, thereby amplifying lung tissue injury (111). Collectively, these pathways drive inflammatory amplification and cell death programs across multiple innate immune cell populations, ultimately reshaping the pulmonary immune microenvironment (112).

An additional upstream regulatory mechanism that may be relevant to EV-based modulation of the cGAS–STING pathway is TRIM29. TRIM29 has been implicated in infection-related lung injury and has been shown to negatively regulate innate immune responses in the lung (97). Mechanistically, TRIM29 can promote K48-linked ubiquitination and degradation of STING (113), thereby suppressing STING-dependent type I interferon signaling. Given the emerging role of EVs in regulating inflammatory signaling through the transfer of miRNAs, siRNAs, and other bioactive cargoes, it is plausible that EVs may alleviate acute lung injury, at least in part, by modulating TRIM29 expression and consequently influencing cGAS–STING pathway activity. However, direct evidence demonstrating that EVs target the TRIM29–STING axis in sepsis-associated ALI is currently lacking, and this possibility should therefore be considered a mechanistically relevant but still speculative direction for future investigation.

**Figure 12:**
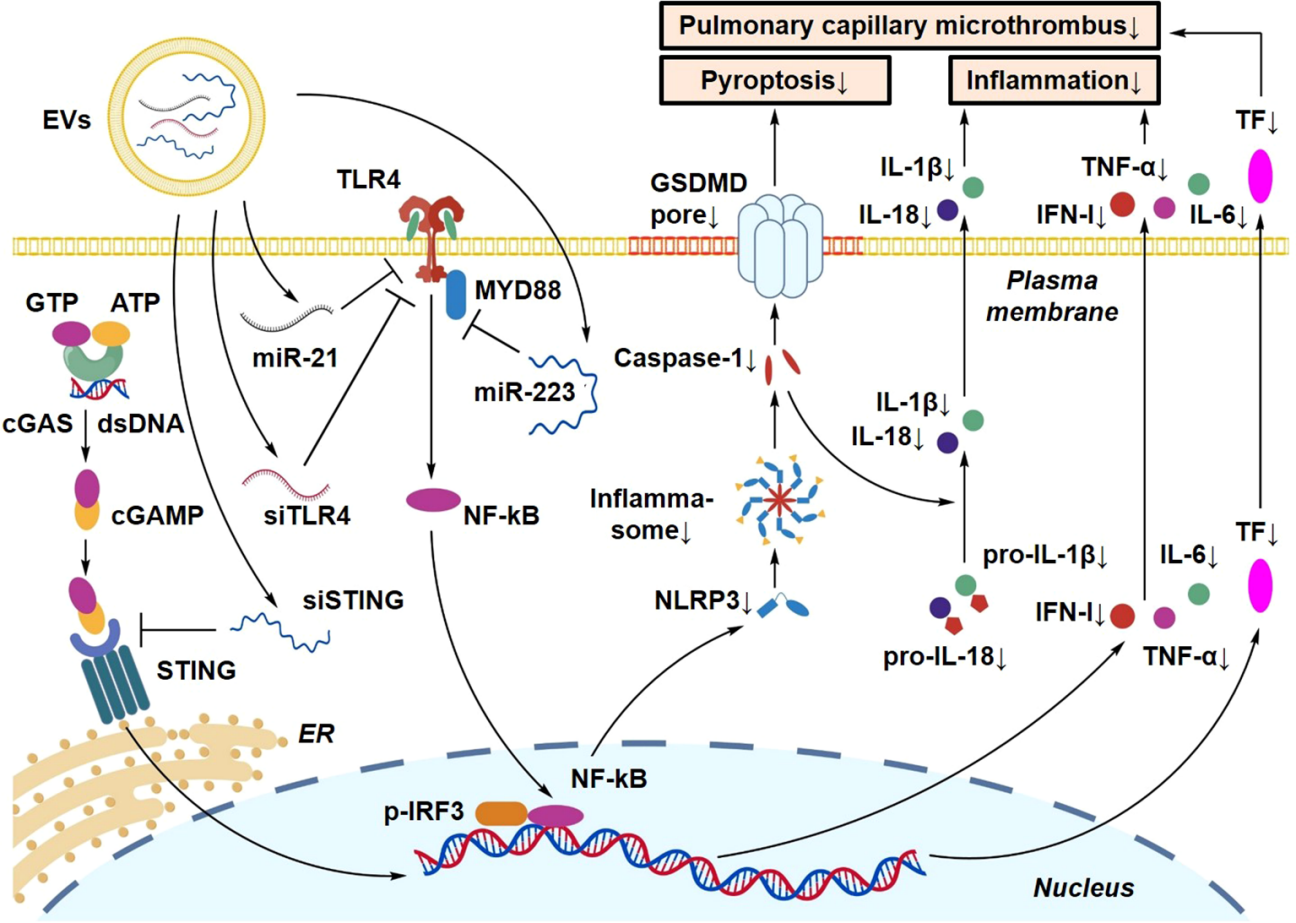
Mechanistic illustration of EV-targeted modulation of the TLR4/NLRP3/cGAS-STING signaling axes. This figure details how EV-delivered cargoes (siRNA, miRNA, or inhibitors) specifically intervene at key nodes of inflammatory pathways. By blocking TLR4, inhibiting NLRP3 assembly, or suppressing STING activation, EVs dampen the excessive inflammatory response.

Recent advances in biomimetic engineering have enabled the integration of small interfering RNA (siRNA) and other nucleic acids into EV-like delivery systems to achieve targeted pathway inhibition. One early study demonstrated that pulmonary delivery of siRNA targeting TLR4 significantly reduced inflammatory responses and neutrophil infiltration in experimental lung injury models (114). Similarly, hucMSC-derived sEVs have been reported to deliver miR-223-3p to lung tissue and attenuate inflammatory injury in ALI (115). In addition, EV-mimetic nanovesicles loaded with anti-miR-155 oligonucleotides have shown anti-inflammatory effects and improved lung injury in LPS-induced ALI models, further supporting the potential of nucleic acid-loaded biomimetic EV systems for pathway-oriented therapeutic intervention (116). Collectively, these findings indicate that EV-based delivery platforms can be engineered to modulate key inflammatory signaling pathways, providing a potential strategy for immune remodeling in sepsis-associated ALI (**Figure 12**).

## 5 Pulmonary targeting and translational considerations

In sepsis-associated ALI, the development of EV-based therapeutic strategies should be interpreted with caution and grounded in current preclinical evidence. Sepsis is characterized by rapid disease progression, dynamic transitions between hyperinflammatory and immunosuppressive states, considerable temporal heterogeneity, and substantial inter-patient variability. These features may significantly influence the timing, dosing, and therapeutic efficacy of EV-mediated immune modulation. In addition, several barriers to clinical implementation remain, including scalable manufacturing, reproducible product characterization, control of biodistribution, immunocompatibility, comprehensive safety evaluation (e.g., potential pro-inflammatory or pro-coagulant effects), and regulatory compliance. The following sections summarize current pulmonary targeting strategies, as well as key considerations related to biosafety, manufacturing, and the translation of preclinical findings into clinical applications.

### 5.1 Active targeting: surface modification and specific recognition

Active targeting strategies aim to enhance targeting to inflamed lung tissue by decorating the surface of EVs with specific ligands or antibodies (117, 118). Pulmonary targets such as SP-A and Siglecs have been explored as targeting sites to improve lung-selective delivery. Notably, SP-A–functionalized engineered EVs loaded with anti-inflammatory cytokines (e.g., IL-4/IL-10) showed improved intrapulmonary retention and attenuated inflammation in murine ALI models (95, 119). Using phage display technology, peptide ligands with high specificity for pulmonary receptors have been identified and fused with EV membrane proteins, generating modified EVs with enhanced lung-targeting capacity (117, 120). In addition, loading regulatory microRNAs such as miR-26a-5p into adipose-derived EVs has been reported to modulate the pulmonary inflammatory microenvironment and inhibit NF-κB signaling, resulting in pronounced anti-inflammatory effects in ALI models (121). Animal studies further demonstrated increased lung accumulation and improved anti-inflammatory efficacy of these engineered EV systems (122).

Passive targeting relies on pathological features of inflamed tissues, including increased vascular permeability and impaired lymphatic drainage, which facilitate nanoparticle accumulation through the enhanced permeability and retention (EPR) effect (123). Due to their nanoscale size and intrinsic biocompatibility, EVs can passively accumulate in inflamed lung tissue via the EPR effect (124). However, the EPR effect shows considerable interindividual variability and often displays limited efficiency in clinical settings. To improve passive targeting, researchers have attempted to optimize EV physicochemical properties, such as particle size, surface charge, and hydrophilicity, to enhance tissue penetration and retention within inflamed regions (125).

The route of EV administration is another critical determinant of pulmonary delivery efficiency and therapeutic outcomes. Intravenous injection is commonly used for systemic delivery but frequently results in preferential accumulation of EVs in organs such as the liver and spleen, which limits lung-targeting efficiency (126). In contrast, inhalation-based delivery enables direct deposition of EVs into the lungs, increasing local therapeutic concentrations while reducing systemic exposure (127). Recent studies have developed aerosolized inhalation systems for delivering engineered EVs to the lungs, demonstrating favorable pulmonary distribution and therapeutic outcomes in animal models (127, 128). For example, Mirsanei and colleagues evaluated the deposition efficiency, biological activity, and local immune responses associated with aerosolized MSC-derived sEVs in ALI models, supporting their potential translational value (129). Nevertheless, EVs derived from different cellular sources or produced using distinct engineering approaches may exhibit substantial variability in pulmonary delivery performance. In addition, aerosolization parameters and EV physicochemical characteristics jointly influence deposition patterns within the respiratory tract and the preservation of biological activity. The stability and functional integrity of EVs during nebulization require further systematic investigation (129, 130).

### 5.2 Biosafety, manufacturing, and regulatory considerations

The immunogenicity of EVs primarily arises from proteins and glycosylation patterns on their membrane surfaces (131). In addition to immunogenicity, emerging evidence suggests that certain EV populations may exert unintended biological effects depending on their cellular origin and molecular cargo. For instance, EVs derived from activated immune or endothelial cells may carry pro-inflammatory mediators capable of amplifying inflammatory signaling. Furthermore, EV-associated surface molecules may potentially trigger off-target immune activation in recipient cells. Some studies have also suggested that EVs can carry pro-coagulant factors such as tissue factor, which may contribute to immunothrombosis under inflammatory conditions. These findings highlight the importance of carefully evaluating EV source, purification strategy, and cargo composition to minimize potential adverse effects during therapeutic development. Heterologous EVs may also induce immune responses that limit their clinical applicability (132). Consequently, rigorous immunogenicity assessment is essential, and the use of autologous or carefully selected allogeneic sources may reduce the risk of immune rejection (133). In addition, EV purification processes must effectively remove potential endotoxins and other contaminants to ensure biosafety (134).

Large-scale production of EVs faces challenges related to cargo consistency, particle purity, and scalable manufacturing (135). Currently, EV isolation and purification largely rely on ultracentrifugation and size-exclusion chromatography; however, alternative approaches such as polymer precipitation or microfluidic-based techniques can produce EV populations with differing purity, cargo composition, and biological activity. This methodological variability may significantly influence experimental outcomes and complicate cross-study comparisons (136). In addition, rigorous EV characterization remains essential. Parameters including particle size distribution, surface marker expression, and cargo composition should be systematically evaluated according to established guidelines, such as the Minimal Information for Studies of Extracellular Vesicles (MISEV) recommendations, to improve reproducibility and interpretability of EV-based studies. Another challenge relates to dose standardization. EV doses are frequently reported using different metrics, including particle number, total protein content, or source cell equivalents, which complicates comparisons across studies and may contribute to inconsistent biological responses. Furthermore, EV cargo composition can vary substantially depending on the physiological state and origin of donor cells, introducing intrinsic heterogeneity that may influence immune regulatory effects. To support industrial-scale production, high-efficiency separation technologies, including microfluidics-based platforms, and standardized manufacturing workflows are required to ensure batch consistency and reliable quality control (137).

Within the regulatory frameworks of the U.S. Food and Drug Administration (FDA) and the International Council for Harmonisation of Technical Requirements for Pharmaceuticals for Human Use (ICH), chemistry, manufacturing, and controls (CMC) represent a central component of regulatory submissions for EV-based biologics. CMC documentation encompasses raw material management, cargo stability, manufacturing consistency, and product quality control (138). In recent years, regulatory agencies, including the FDA and the European Medicines Agency (EMA), have updated their perspectives on cell-based and EV-derived therapeutics, recommending CMC strategies aligned with established biologics standards and emphasizing requirements for batch consistency, potency evaluation, and immunogenicity assessment (138, 139). As a novel class of biological products, EVs require comprehensive CMC documentation covering manufacturing processes, quality control procedures, and stability studies (140). However, regulatory guidance specific to EV-based therapies remains limited, highlighting the need for ongoing communication between developers and regulatory authorities to establish compliant manufacturing strategies and ensure product safety and efficacy (141, 142).

### 5.3 Bridging preclinical models and clinical translation

Murine models are widely used in studies of ALI and its severe manifestation, ARDS; however, important anatomical and immunological differences between mice and humans may limit the translational relevance of EV-based therapies (143). For example, murine lungs differ markedly from human lungs in alveolar structure and immune cell composition. In addition, murine neutrophils display substantial differences from their human counterparts in phenotype, effector molecule expression, and response magnitude, which may influence the mechanistic interpretation of ALI and the extrapolation of therapeutic outcomes (144). Mice also exhibit significantly lower sensitivity to LPS than humans, introducing further uncertainty when translating findings from animal models to clinical settings (145, 146). These limitations highlight the importance of considering interspecies differences when designing preclinical studies and underscore the need for model systems that better recapitulate human pulmonary physiology and immune responses.

Advances in organoid and organ-on-a-chip technologies have provided promising alternatives for modeling lung disease and evaluating nanomedicine safety. Lung organoids generated from human epithelial cells can reproduce key aspects of alveolar structure and function, and immune-enhanced organoid models incorporating macrophages and other immune cells allow *in vitro* simulation of infection- or LPS-induced inflammatory responses (147, 148). Recent developments have produced platforms capable of modeling immune cell migration, including neutrophil and T cell recruitment, thereby offering useful tools for investigating EV-mediated immune regulation in inflammatory microenvironments (149). Second-generation lung-on-a-chip systems incorporate stretchable biomembranes composed of collagen and elastin, enabling simulation of alveolar mechanics under dynamic physiological conditions (150). These devices can reproduce the air–blood barrier and endothelial–epithelial interfaces using microfluidic technology, allowing more accurate modeling of vascular leakage and pulmonary inflammation (151). For example, lung-on-a-chip models have been used to evaluate the protective effects of MSC-derived EVs on the alveolar–capillary barrier, demonstrating restoration of transepithelial electrical resistance and improved expression of junctional proteins (152).

EV-based therapeutics are currently undergoing clinical investigation worldwide. According to ClinicalTrials.gov, more than 60 clinical studies related to MSC-derived EVs were registered between 2014 and 2024, covering cardiovascular, inflammatory, and pulmonary diseases, with several advancing to Phase I or II trials (153). Although no EV-based therapy has yet received approval from the U.S. FDA for the treatment of ALI, regulatory frameworks for EV-based biologics are gradually becoming more defined (138). To initiate investigational new drug (IND) applications, developers are required to provide comprehensive documentation including CMC, preclinical safety evaluation, and efficacy data (154). Recent analyses suggest that IND preparation for EV-based products should include several core components, such as source cell traceability, purification consistency, carrier stability, bioactivity standards, and structured CMC documentation. In addition, early and continuous communication with regulatory authorities has been recommended to facilitate regulatory review and accelerate clinical progression (154). Regulatory agencies in Europe, the United States, and China are also gradually establishing frameworks specific to EV-based therapeutics, providing an essential policy foundation for future clinical translation (141, 155).

Pulmonary targeting strategies and translational pathways are critical determinants for the clinical success of EV-based therapies. From a delivery perspective, combining active targeting strategies with localized administration may improve therapeutic efficiency (129). From a biosafety perspective, comprehensive quality control systems are required to address potential immunogenicity, purification residues, and manufacturing scale-up challenges (138). In translational research, increasing emphasis should be placed on human-relevant models, including organoids and lung-on-a-chip systems, while progressively generating key IND-enabling data and adapting to evolving regulatory requirements. Notably, lung organoid models have been shown to preserve host-specific immune networks under inflammatory conditions, including functional indicators of alveolar macrophages and CD8^+^ T cells, making them valuable platforms for investigating EV-mediated immune regulation (156). Ultimately, multidisciplinary collaboration among medical, engineering, and regulatory fields will be essential for advancing EV-based technologies from laboratory research toward clinical application (154).

## 6 Future directions and multidisciplinary integration: toward precision immune intervention platforms

Sepsis-associated ALI, characterized by pronounced immunological heterogeneity, is unlikely to be effectively managed through a single therapeutic modality. As hybrid platforms integrating immune regulation with targeted delivery, EV-based technologies may benefit from advances in multidisciplinary collaboration, system-level integration, and personalized therapeutic design. Progress along these directions could facilitate the development of precision immune intervention platforms and promote the translation of EV-based strategies from experimental research to clinical application.

### 6.1 Artificial intelligence-assisted EV design and target identification

AI is increasingly being applied in the design and optimization of EV-based therapeutic systems. Applications include miRNA combination screening, modeling of cell–cell communication networks, and structural optimization of delivery carriers, where notable improvements in efficiency have already been reported (157, 158). Integrative bioinformatic and machine-learning analyses of transcriptomic and single-cell datasets can identify key molecular signatures associated with the progression of sepsis-associated ALI, thereby providing candidate targets for EV cargo selection and delivery strategies (159). Recent studies have also explored AI-driven models that associate EV cargo composition with functional outcomes. By integrating multi-omics datasets, including transcriptomic and miRNA profiles, AI-based approaches may help predict potential target pathways and biological responses to EV-delivered molecules, supporting the rational design of engineered EV systems (160, 161). In addition, deep learning methods have been developed for high-throughput profiling of multiple miRNAs within individual exosomes, providing computational tools to improve miRNA selection and loading strategies (162).

**Figure 13:**
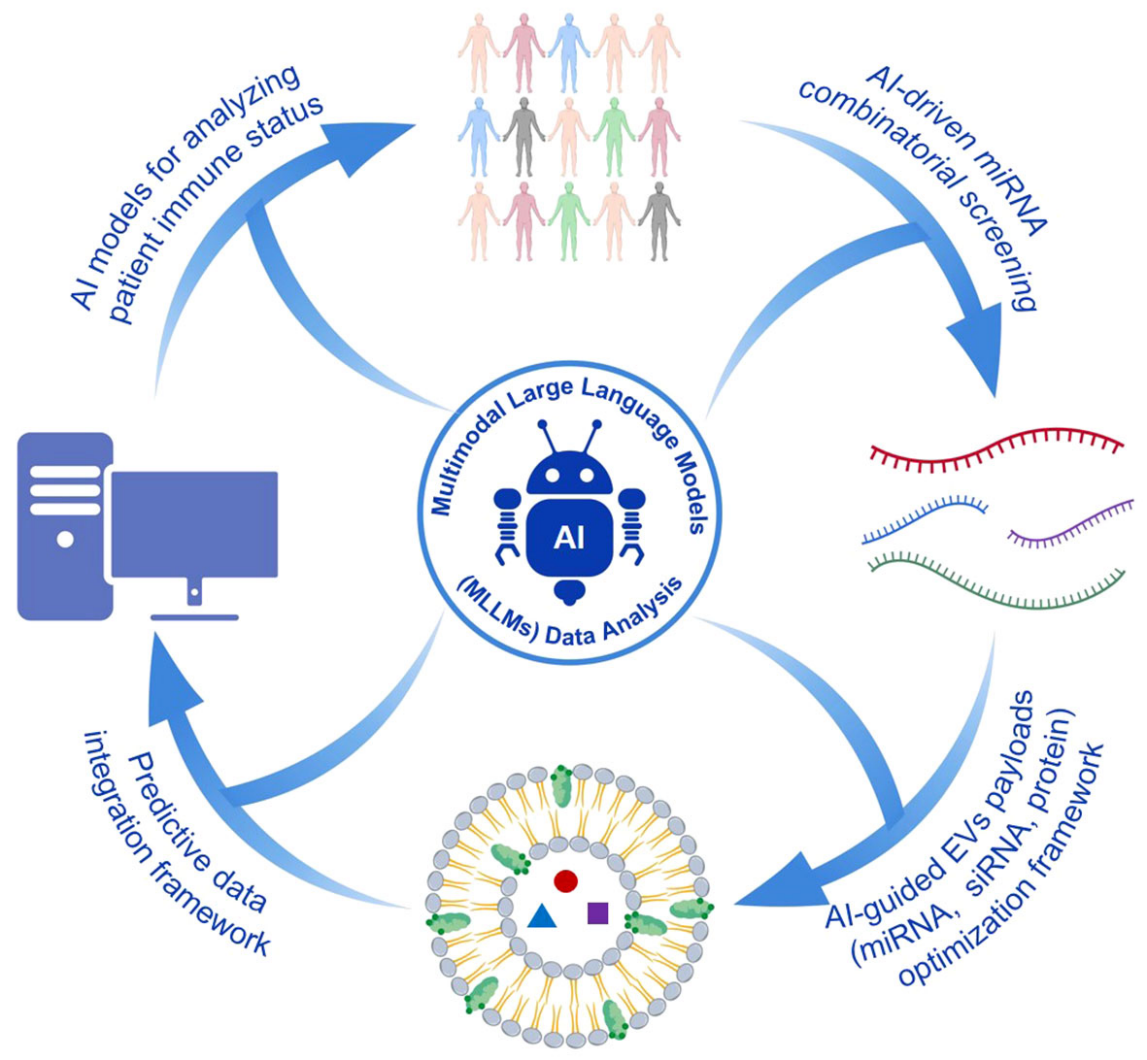
AI-assisted EV design workflow and closed-loop immune intervention framework. Artificial intelligence algorithms analyze multi-omics data to identify optimal therapeutic targets and predict EV cargo behavior. This facilitates the design of personalized EV therapies and creates a closed-loop system for precise immune modulation.

By learning from large-scale transcriptomic datasets related to sepsis, AI can predict interactions between miRNA-loaded EVs and key inflammatory pathways, such as TLR and NF-κB signaling (163, 164). This capability may facilitate early prediction of immune response patterns, thereby reducing the risk of excessive immune activation or unintended immune tolerance (165). Beyond improving design efficiency, AI-based frameworks may support the development of patient-specific EV therapeutic strategies. By integrating patient-derived transcriptomic, miRNA, and exosomal datasets, AI systems could establish closed-loop workflows that link immune state modeling, EV cargo design, and response prediction, thereby providing a technical basis for personalized immune intervention (**Figure 13**) (160, 166).

### 6.2 Multimodal synergistic strategies: from immune regulation to integrated intervention

Under complex pathological conditions such as sepsis-associated ALI, targeting a single immune pathway is often insufficient to interrupt the interconnected cycle of infection, inflammation, and tissue injury (167). Owing to their intrinsic cargo-delivery capacity, EV-based platforms are well-suited for co-loading multiple therapeutic agents, including anti-inflammatory molecules, antimicrobial factors, and signaling pathway modulators, thereby enabling the development of multimodal therapeutic systems. For instance, an EV-encapsulated nanotherapeutic strategy delivering Clara cell secretory protein 16 (CC16) demonstrated anti-inflammatory and lung-protective effects at reduced doses in experimental ALI models (168). In addition, MSC-EVs, as cell-free formulations, offer potential advantages in minimizing immunogenicity and controlling infusion-related risks (169).

**Figure 14:**
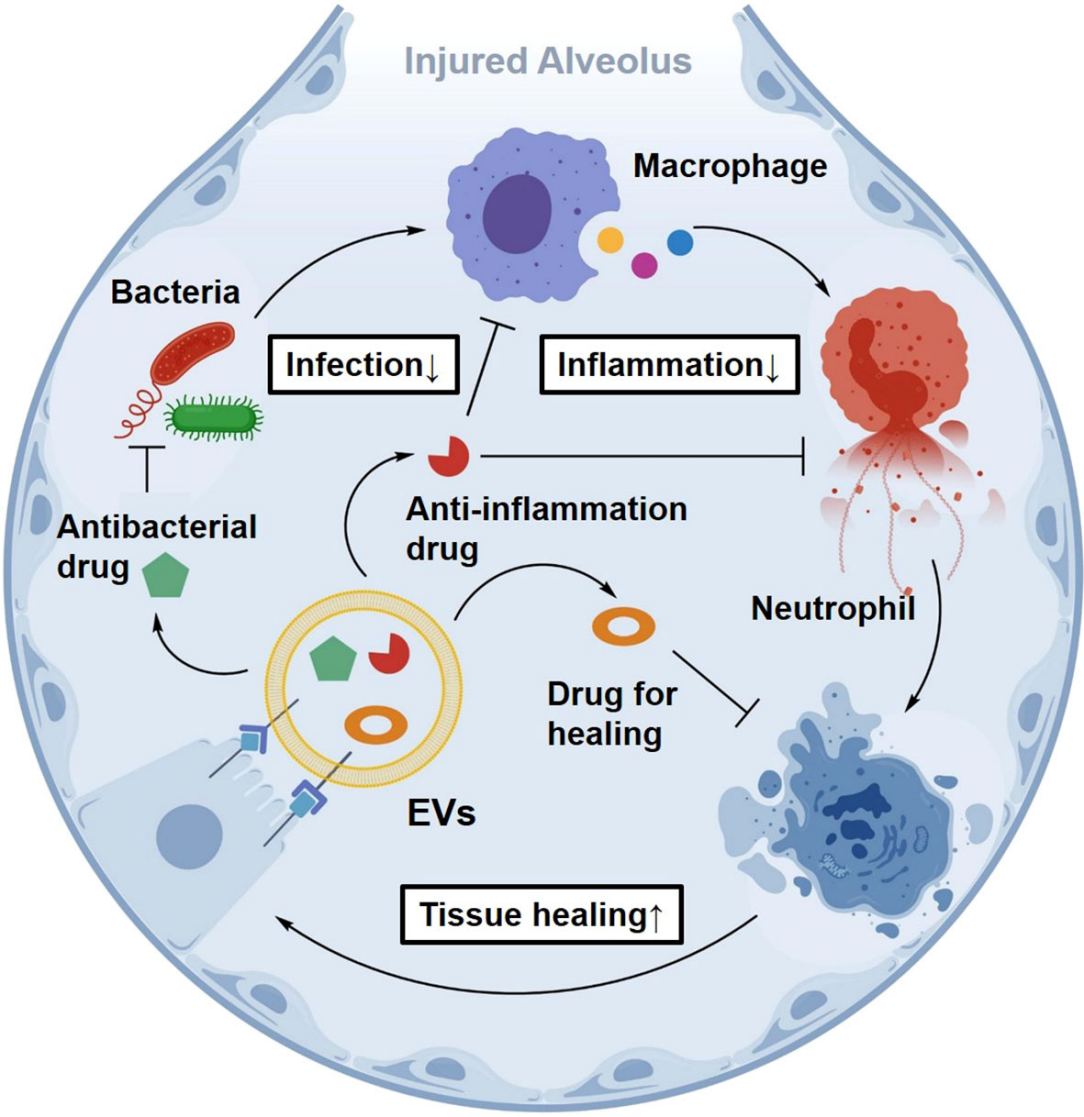
Schematic overview of multimodal biomimetic EV system construction and composite cargo loading. An illustration of advanced EV systems capable of co-loading diverse agents (e.g., anti-inflammatory drugs, antibiotics, and nucleic acids). This multimodal approach allows for simultaneous targeting of infection, inflammation, and tissue repair.

Collectively, this approach may support the development of a closed-loop intervention paradigm integrating targeting, regulation, and repair, thereby addressing some limitations of conventional single-target therapies (**Figure 14**).

### 6.3 Construction of intelligent integrated diagnostic-therapeutic systems

Integrated diagnostic–therapeutic platforms represent an important direction in the evolution of EV-based technologies. By incorporating imaging probes together with therapeutic cargoes, such systems enable real-time monitoring of EV biodistribution and treatment responses, forming a closed-loop strategy that links targeting, cargo release, and feedback monitoring (170). For example, near-infrared imaging probes and magnetic resonance imaging contrast agents have been successfully integrated into EV platforms, enabling simultaneous visualization of pulmonary inflammation and therapeutic intervention (171). Furthermore, the “SMART-EVs” platform integrates imaging quantum dots, TLR4-targeting siRNA, and Siglec-1-specific antibodies within a single EV construct, providing a proof-of-concept paradigm for precise, image-guided intervention at inflammatory sites (172–174).

**Figure 15:**
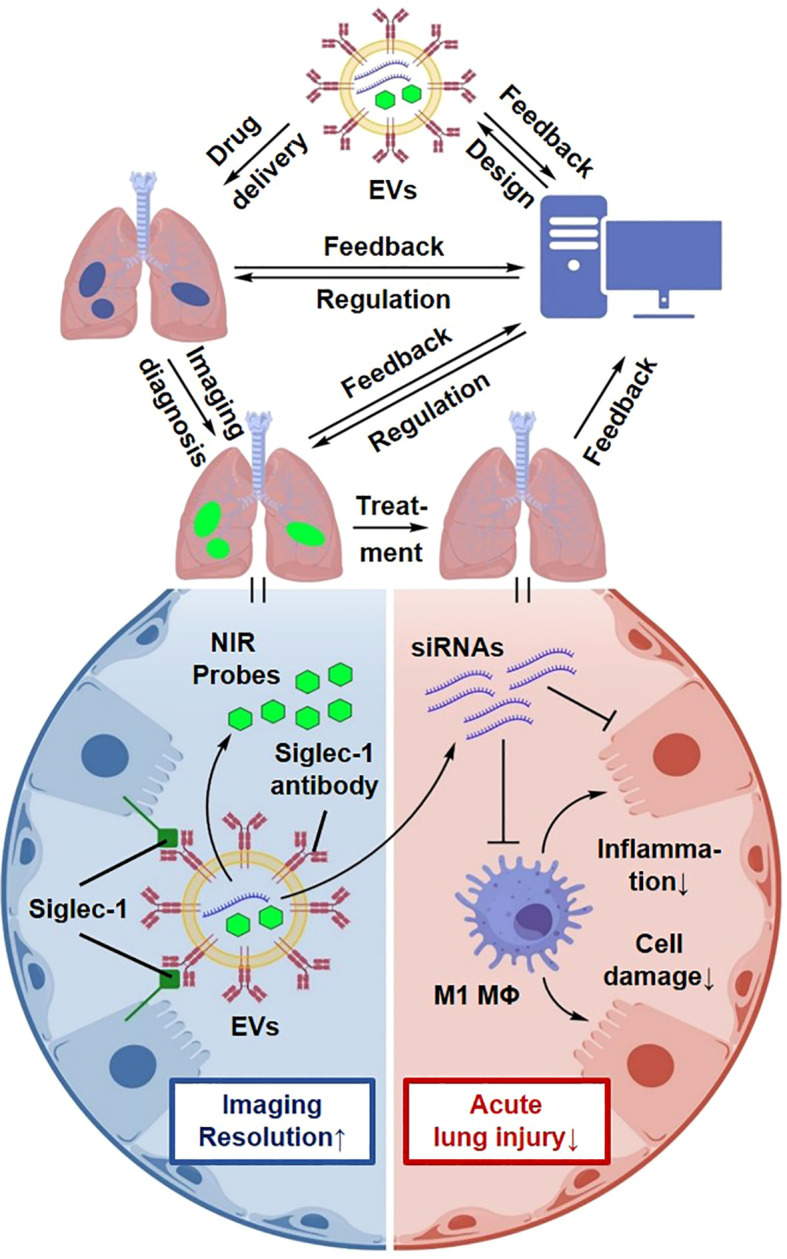
Conceptual illustration of an intelligent integrated diagnostic-therapeutic EV platform. A futuristic concept of “theranostic” EVs that combine therapeutic functions with imaging probes (e.g., quantum dots, MRI contrast agents). This platform enables real-time visualization of drug delivery and monitoring of treatment efficacy in sepsis-associated ALI.

Beyond therapeutic applications, such integrated platforms may also serve as monitoring tools in critical care settings, enabling assessment of pneumonia progression and real-time evaluation of anti-inflammatory responses (**Figure 15**).

### 6.4 Limitations and challenges

Despite the promising potential of EV-based biomimetic nanosystems for the treatment of sepsis-associated ALI, several important limitations should be considered. First, most of the available evidence is derived from preclinical rodent models. Experimental systems such as LPS-induced injury or cecal ligation and puncture cannot fully reproduce the heterogeneity, temporal progression, and comorbid conditions observed in human sepsis, which contributes to a persistent translational gap. Second, although various engineering strategies have been proposed, the lack of standardized protocols for EV isolation, purification, characterization, and storage remains a major obstacle for reproducibility and large-scale production. In addition, EV cargo composition may vary depending on the physiological state and origin of donor cells, introducing variability in therapeutic outcomes. Third, the biosafety profile of engineered EV systems requires further evaluation. Issues such as potential immunogenicity, off-target biodistribution, and long-term biological effects remain insufficiently characterized, particularly in critically ill patient populations. Finally, while multidisciplinary approaches offer new opportunities for innovation, the cost-effectiveness and regulatory pathways for personalized EV-based therapies remain significant challenges for widespread clinical implementation. Addressing these issues through standardized methodologies, international collaboration, and improved human-relevant models will be essential for future progress.

### 6.5 Building a “precision immune intervention” platform: future directions for multidisciplinary integration

AI–assisted design, multimodal therapeutic strategies, integrated diagnostic–therapeutic systems, and personalized treatment approaches represent key components supporting the next generation of EV-based technologies. However, advancing these strategies independently may not achieve substantial progress. Integrating these innovations into a unified “precision immune intervention” framework may therefore represent a central objective for future development. Such a platform would ideally incorporate four core capabilities:

1. Intelligent design capability: leveraging AI to identify individual-specific targets from multi-omics datasets and to guide rational EV cargo combinations (157);
2. Multimodal intervention capability: integrating immune regulation with anti-infective and other therapeutic functions to generate synergistic effects (109);
3. Targeted visualization capability: enabling real-time monitoring of delivery routes and therapeutic outcomes through molecular imaging components (175);
4. Manufacturing and translational capability: establishing scalable systems that support standardized production, rigorous quality control, and regulatory compliance (176).

Looking forward, deeper integration across medicine, bioengineering, materials science, data science, and clinical practice will be critical for refining this framework. This multidisciplinary collaboration may facilitate the transition of immune intervention strategies for sepsis-associated ALI from generalized modulation toward more individualized and precisely targeted therapeutic approaches.

## 7 Conclusion

Sepsis-associated ALI and ARDS remain among the most challenging syndromes in critical care medicine. Their pathogenesis is characterized by a complex cycle involving dysregulated innate immune responses, excessive inflammatory activation, and impaired tissue repair. In recent years, EVs and EV-based biomimetic nanocarriers have emerged as promising nanoscale platforms with intrinsic capabilities for immune modulation and targeted delivery, offering new perspectives for the treatment of this multifactorial disease.

Guided by a four-dimensional framework encompassing mechanisms, immune regulation, engineering strategies, and translational considerations, this review systematically summarizes the immunopathological basis of sepsis-associated ALI and examines the emerging roles of EVs in regulating key innate immune components, including neutrophil activity, macrophage polarization, and dendritic cell–mediated antigen presentation. In addition, recent advances in biomimetic engineering and pulmonary-targeted delivery strategies were integrated to illustrate how EV-based systems may bridge mechanistic insights with preclinical therapeutic development. Compared with previous reviews focusing on isolated aspects, this work proposes an EV-centered conceptual framework for immune remodeling and incorporates emerging technologies, including AI–assisted design, integrated diagnostic–therapeutic systems, and personalized engineering, into a broader systems-level perspective.

At present, MSC-EVs and their engineered derivatives have demonstrated encouraging immunomodulatory effects in multiple experimental models of ALI. However, successful clinical translation will require overcoming several key challenges, including scalable and standardized manufacturing, consistent product characterization, long-term safety validation, and the establishment of robust CMC systems.

Looking forward, multidisciplinary collaboration will play a pivotal role in advancing EV-based technologies toward clinical application. Future development directions, including AI-assisted design, multimodal therapeutic strategies, imaging-guided intervention, and standardized regulatory frameworks, may collectively support the evolution of EV-based platforms for immune modulation. With continued mechanistic investigation and translational optimization, EV-based biomimetic systems may contribute to future immune-intervention strategies for sepsis-associated ALI. Nevertheless, critical issues such as controllable biodistribution, immunocompatibility and safety assessment (e.g., potential pro-inflammatory or pro-coagulant effects), and regulatory readiness must be carefully addressed before clinical implementation can be realistically achieved.

## Glossary

AI: Artificial Intelligence
ALI: Acute Lung Injury
ARDS: Acute Respiratory Distress Syndrome
CMC: Chemistry Manufacturing and Controls
DAMPs: Damage-Associated Molecular Patterns
DC: Dendritic Cell
EPR: Enhanced Permeability and Retention
EVs: Extracellular Vesicles
FDA: U.S. Food and Drug Administration
hucMSC: Human Umbilical Cord-Derived Mesenchymal Stem Cell
ICUs: Intensive Care Units
IL-1: Interleukin-1
IL-1β: Interleukin-1 Beta
IL-6: Interleukin-6
IL-10: Interleukin-10
IND: Investigational New Drug
iNOS: Inducible Nitric Oxide Synthase
JAK: Janus Kinase
lncRNAs: Long Non-Coding RNAs
LPS: Lipopolysaccharide
MHC-II: Major Histocompatibility Complex Class II
MPO: Myeloperoxidase
MSC-EVs: Mesenchymal Stem Cell-Derived Extracellular Vesicles
MSCs: Mesenchymal Stem Cells
miRNAs: MicroRNAs
NETosis: Neutrophil Extracellular Trap Formation
NETs: Neutrophil Extracellular Traps
PAMPs: Pathogen-Associated Molecular Patterns
PD-L1: Programmed Death-Ligand 1
PEI: Polyethylenimine
PLGA: Poly(Lactic-Co-Glycolic Acid)
PRRs: Pattern Recognition Receptors
ROS: Reactive Oxygen Species
sEVs: Small Extracellular Vesicles
siRNA: Small Interfering RNA
SIRS: Systemic Inflammatory Response Syndrome
TGF-β: Transforming Growth Factor-Beta
TNF-α: Tumor Necrosis Factor-Alpha
Tregs: Regulatory T Cells.
